# RNA editing in nascent RNA affects pre-mRNA splicing

**DOI:** 10.1101/gr.231209.117

**Published:** 2018-06

**Authors:** Yun-Hua Esther Hsiao, Jae Hoon Bahn, Yun Yang, Xianzhi Lin, Stephen Tran, Ei-Wen Yang, Giovanni Quinones-Valdez, Xinshu Xiao

**Affiliations:** 1 Department of Bioengineering,; 2 Department of Integrative Biology and Physiology, University of California Los Angeles, Los Angeles, California 90095, USA;; 3 Bioinformatics Interdepartmental Program, University of California Los Angeles, Los Angeles, California 90095, USA;; 4 Molecular Biology Institute, University of California Los Angeles, Los Angeles, California 90095, USA

## Abstract

In eukaryotes, nascent RNA transcripts undergo an intricate series of RNA processing steps to achieve mRNA maturation. RNA editing and alternative splicing are two major RNA processing steps that can introduce significant modifications to the final gene products. By tackling these processes in isolation, recent studies have enabled substantial progress in understanding their global RNA targets and regulatory pathways. However, the interplay between individual steps of RNA processing, an essential aspect of gene regulation, remains poorly understood. By sequencing the RNA of different subcellular fractions, we examined the timing of adenosine-to-inosine (A-to-I) RNA editing and its impact on alternative splicing. We observed that >95% A-to-I RNA editing events occurred in the chromatin-associated RNA prior to polyadenylation. We report about 500 editing sites in the 3′ acceptor sequences that can alter splicing of the associated exons. These exons are highly conserved during evolution and reside in genes with important cellular function. Furthermore, we identified a second class of exons whose splicing is likely modulated by RNA secondary structures that are recognized by the RNA editing machinery. The genome-wide analyses, supported by experimental validations, revealed remarkable interplay between RNA editing and splicing and expanded the repertoire of functional RNA editing sites.

The maturation of eukaryotic mRNAs involves an intricate series of molecular processes that modify newly synthesized RNA molecules, such as 5′ capping, splicing, RNA editing, and polyadenylation (Bentley 2014). These RNA processing steps play essential roles in eukaryotic gene expression (Nishikura 2015; Martins and Fåhraeus 2017). Recent studies have enabled substantial progress in understanding the regulation and function of each RNA processing step. However, it is expected that extensive crosstalk exists between these molecular processes (Mitchell and Parker 2014; Hsiao et al. 2016b), and such crosstalk remains poorly understood.

Among the various RNA processing steps, RNA editing and RNA splicing share the largest overlap in time and space during mRNA maturation (Bentley 2014). Catalyzed by the Adenosine Deaminases Acting on RNA (ADARs), adenosine-to-inosine (A-to-I) editing is the most prevalent type of RNA editing in mammals (Nishikura 2010; Bazak et al. 2014). Inosine is recognized as guanosine (G) by most cellular machineries. As a result, A-to-I editing could alter regulatory motifs, RNA-protein interactions, or RNA secondary structures, all of which play important roles in modulating RNA processing (Raitskin et al. 2001; Rieder and Reenan 2012; Washburn and Hundley 2016). Indeed, there are a handful of well-documented examples in which A-to-I editing affects alternative splicing (Higuchi et al. 1993, 2000; Rueter et al. 1999; Beghini et al. 2000; Reenan et al. 2000; Bratt and Ohman 2003; Flomen et al. 2004; Laurencikiene et al. 2006; Lev-Maor et al. 2007; Schoft et al. 2007; Agranat et al. 2010; Penn et al. 2013). However, the specific mechanisms for some of these events remain unclear.

Creation or elimination of splice sites and branch points by RNA editing was shown to explain several editing-dependent splicing events (Rueter et al. 1999; Beghini et al. 2000; Flomen et al. 2004; Laurencikiene et al. 2006; Lev-Maor et al. 2007; Schoft et al. 2007). Previous studies also demonstrated that A-to-I editing of *Alu* repeats enabled exonization of the noncoding sequences (e.g., via creation of a 3′ splice site), suggesting that RNA editing may be an important means for exon evolution (Athanasiadis et al. 2004; Lev-Maor et al. 2007; Möller-Krull et al. 2008). Another mechanism through which RNA editing may influence splicing is by alteration of RNA secondary structures (Rieder and Reenan 2012). Because ADAR proteins recognize double-stranded RNAs (dsRNAs), substitution of adenosines by inosines may alter the stability of the RNA structures and influence the accessibility of splice sites (Morse et al. 2002; Blow et al. 2004; Levanon et al. 2004; Mazloomian and Meyer 2015). In general, ADAR and splicing machineries may act on the same dsRNA substrates, leading to the interplay between these two processes (Reenan et al. 2000; Rieder et al. 2013). However, structure-mediated splicing modulation by RNA editing or ADAR binding is relatively poorly understood.

In addition to editing-dependent splicing events, the reverse could also be true, that is, splicing affects the outcomes of RNA editing. For example, intron removal could determine the availability of editing complementary sequences (ECSs) that are required to form dsRNA substrates for editing (Levanon et al. 2005). Thus, RNA splicing efficiency could play a key role in regulating RNA editing levels (Licht et al. 2016). In addition, the interaction between ADARs and splicing factors may also fine-tune editing efficiency (Tariq et al. 2013). These studies have further demonstrated the importance of deciphering the interplay between RNA editing and splicing to achieve a better understanding of the regulation of both processes.

On the global scale, the prevalence of RNA editing events that affect alternative splicing and the associated mechanisms remain unclear. If this functional pathway is widespread, RNA editing must occur cotranscriptionally and prior to the completion of most splicing events. Previous studies provided evidence of cotranscriptional editing by showing that the C-terminal domain of Pol II-facilitated site-specific editing, and that editing occurs prior to splicing in several genes (Laurencikiene et al. 2006; Ryman et al. 2007). In addition, a recent global study showed that A-to-I editing modifies nascent RNAs cotranscriptionally in *Drosophila* (Rodriguez et al. 2012). Moreover, reduction of ADAR expression in both *Drosophila* and human cells induced global splicing changes (Agrawal and Stormo 2005; Solomon et al. 2013; St Laurent et al. 2013), although the molecular mechanisms are unknown.

Despite the aforementioned progress, the kinetic timing of RNA editing during mRNA maturation in human cells remains to be characterized. Furthermore, a global understanding of the influence of RNA editing on splicing is still lacking. Our study aims to address these questions. We first elucidated the timing of RNA editing during mRNA maturation. The finding that >95% of A-to-I RNA editing occurred cotranscriptionally in nascent RNAs motivated the hypothesis that the impact of RNA editing on splicing is more widespread than previously appreciated. We then focused on investigating two potential mechanisms: (1) splicing alteration by RNA editing in splice site sequences; and (2) splicing modulation by ADAR binding and editing of dsRNA structures. Supported by experimental validations, our study expands the repertoire of RNA editing sites that influence alternative splicing and provides new insights regarding the interplay between these two processes.

## Results

### Subcellular RNA-seq and RNA editing

To examine the timing of RNA editing occurrence during mRNA maturation, we performed cell fractionation (Bhatt et al. 2012) to extract RNA in the chromatin-associated (Ch), nucleoplasmic (Np), and cytoplasmic (Cp) fractions of a human glioblastoma cell line (U87MG). The separation quality of these subcellular fractions was confirmed by Western blot (Supplemental Fig. S1). We obtained RNA-seq data in triplicates, with two types of RNA (polyadenylated and nonpolyadenylated) in the Ch fraction and polyadenylated RNA in the Np and Cp fractions (referred to as ChA^−^, ChA^+^, NpA^+^, and CpA^+^, respectively) (Supplemental Table S1). Read mapping was then carried out using our previously published method, which has superior performance in handling single-nucleotide variants in the reads (Ahn and Xiao 2015).

We observed that the ChA^−^ fraction yielded a large amount of intronic reads, much more abundant than those in the other fractions (Supplemental Fig. S2). This result is consistent with the expectation that ChA^−^ RNA mainly consists of nascent RNA molecules that are not yet fully spliced. The percentage of intronic reads in ChA^+^ was much larger than those in later stages, such as NpA^+^ and CpA^+^, indicating incomplete splicing in the ChA^+^ fraction, which is consistent with previous literature (Bhatt et al. 2012). In contrast, the percentage of reads mapped to exons increased in the order of ChA^−^, ChA^+^, NpA^+^, and CpA^+^. These observations are consistent with the expectation that the four subcellular fractions capture RNA molecules at different stages of their life cycle. Biological replicates showed highly similar results (Supplemental Fig. S3) and were thus combined for the RNA editing analysis described below.

Next, we identified RNA editing sites using our previously published methods (Bahn et al. 2012; Lee et al. 2013; Zhang and Xiao 2015). In addition, to capture A-to-I editing sites that cluster in close proximity, we implemented a pipeline, similar to that in Porath et al. (2014), to identify editing sites in hyperedited regions (Methods). Altogether, we identified about 80,000 to approximately 367,000 editing sites per fraction, with an average of 83% comprising A-to-I and C-to-U types (Supplemental Table S2). It should be noted that ChA^−^ had the most non-A-to-G or non-C-to-T types, which may reflect existence of unknown biology (Wang et al. 2014). Among all the A-to-G sites identified (440,821 in total), 64% and 75% were included in the RADAR (Ramaswami and Li 2014) and REDIportal (Picardi et al. 2017) databases, respectively. Hereafter, we will limit all analyses to the predicted A-to-G editing sites, given that our primary focus is on A-to-I editing.

### More than 95% of A-to-I editing occurs cotranscriptionally prior to polyadenylation

**Figure 1.**
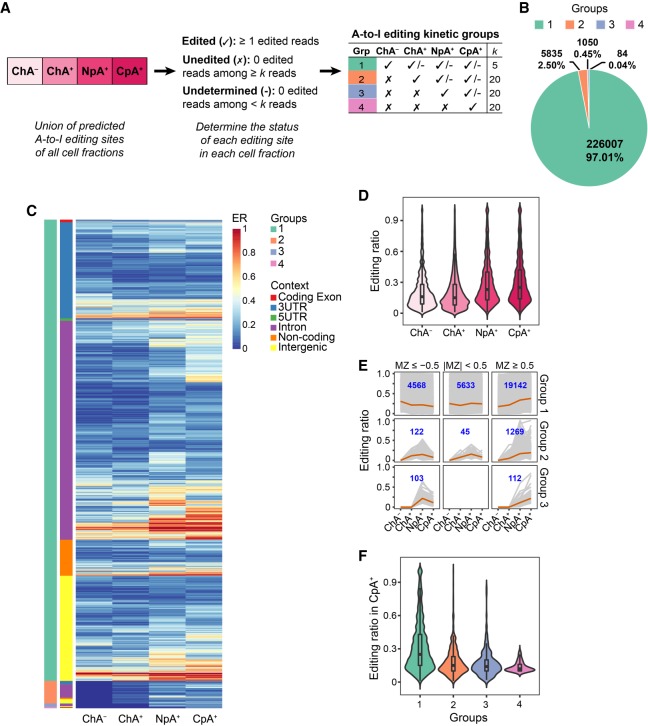
Kinetic groups of RNA editing sites identified via subcellular RNA-seq. (*A*) Categorization of RNA editing sites into four kinetic groups using cell fractionation RNA-seq. Editing sites in each group are labeled as edited (✓), unedited (✗), or undetermined (-) in each subcellular fraction. The read coverage cutoffs of the editing sites (*k*) are illustrated in the table (for details, see Methods). (*B*) Number and percentage of A-to-I RNA editing sites in each editing kinetics group. (*C*) The editing ratios (ER) of each editing site in the four groups. The genomic location of an editing site is shown in color codes. All editing sites included in this analysis were required to have at least five total reads in each fraction (same for panels *D*–*F*). A total of 29,345, 1437, 220, and 84 sites were included for groups 1, 2, 3, and 4, respectively, with group 1 editing sites accounting for 94% of all. (*D*) Editing ratio distribution of the editing sites in *C* (union of all four kinetic groups) in each subcellular fraction. Note that for each fraction, editing ratios of the union of all groups are shown. (*E*) Editing ratios in different subcellular fractions of editing sites in *C* with different ranges of MZ scores. Each gray line represents one editing site. The average editing ratios are highlighted in orange. The number of editing sites in each panel is shown in blue. It should be noted that MZ scores cannot be calculated for sites with constant editing ratios across two consecutive fractions; thus, they were excluded from this analysis. (*F*) Editing ratios of each kinetics group observed in mature mRNAs (CpA^+^).

The subcellular fractionation data allowed us to examine where editing was first observed, thus shedding light on the kinetic timing of RNA editing. We took the union of all editing sites observed in at least one fraction (440,821 in total). Hereafter, we refer to this collection of sites as “editing sites” in general. It is important to note that these editing sites could be edited, unedited, or undetermined (without adequate read coverage) in a specific fraction (Fig. 1A; Methods).

To obtain a detailed view of editing events across subcellular fractions, we enumerated all possible scenarios of an editing site being “edited,” “unedited,” or “undetermined” in each fraction (Supplemental Table S3). We further classified the editing sites into four groups based on their editing status in each fraction (Fig. 1A; Methods). The groups (1–4) included editing sites that were first observed with edited reads in ChA^−^, ChA^+^, NpA^+^, and CpA^+^, respectively. This grouping represents an approximate kinetic timing of editing occurrence. For example, the ChA^−^ group consists of early editing events occurring in nascent RNAs, whereas the CpA^+^ group reflects “late” editing events that were first observed in the later stage of an RNA's life cycle. A total of 232,976 A-to-I editing sites (53% of all) were categorized into one of the four groups (Fig. 1B; Supplemental Fig. S4). The sites that could not be unambiguously classified into these groups were excluded (Supplemental Table S3), which could be due to two main reasons. The first reason is inadequate read coverage in at least one of the cell fractions. In general, if an editing site was “undetermined” due to low read coverage in any fractions prior to the fraction where it was observed with edited reads, this editing site cannot be categorized unambiguously. A total of 170,268 such editing sites were identified and excluded from the grouping. Second, an editing site was excluded if it was “unedited” (with adequate read coverage, but no edited reads) in a later fraction but “edited” in an earlier fraction. A total of 37,577 such sites were identified. These editing sites may be associated with complicated regulatory or functional mechanisms, which should be investigated in future studies.

Among all categorized editing sites, more than 226,000 (97%) were included in group 1 (ChA^−^). This observation suggests that most editing events occur cotranscriptionally in nascent RNA prior to polyadenylation. Since group 4 (CpA^+^-specific) consists of a very small number of editing sites (84 sites total, which is 0.04% of all sites), we excluded this group from further analyses.

To corroborate the aforementioned observed kinetic timing of RNA editing using independent data sets, we analyzed the cell fractionation RNA-seq data from the ENCODE Project in which nuclear poly(A)^−^ (NA^−^), nuclear poly(A)^+^ (NA^+^), and cytosolic poly(A)^+^ (CA^+^) data were obtained from human cell lines (HepG2 and K562) (Djebali et al. 2012) and another ENCODE data set of chromatin-associated (ribo-depleted) and nucleoplasmic (ribo-depleted) RNA-seq of K562 cells (Djebali et al. 2012). These analyses showed that >95% of editing sites occurred in the NA^−^ or chromatin fraction that largely consists of nascent RNAs or spliced introns (Supplemental Fig. S5; Methods). Furthermore, we analyzed additional RNA-seq data that captured nascent RNA using 4sU labeling, a method that is completely different from cell fractionation (Rabani et al. 2014). These data were derived from mouse dendritic cells after lipopolysaccharide (LPS) stimulation. The results demonstrated again that the majority of RNA editing sites occurred in nascent RNAs shortly after their transcription (Supplemental Fig. S6). Together, our analyses showed widespread cotranscriptional RNA editing in human and mouse cells, which is consistent with the previous finding in *Drosophila* (Rodriguez et al. 2012). Furthermore, our data suggest that the vast majority of RNA editing not only occurs cotranscriptionally, but also precedes polyadenylation.

### Comparative analyses of RNA editing sites across subcellular fractions

Next, we carried out a comparative analysis of A-to-I editing in different subcellular fractions. For this purpose, we included editing sites with a total read coverage of at least five in all fractions. The levels of RNA editing differ greatly for different editing sites (Fig. 1C). In each subcellular fraction, editing levels varied from 0.01 to 1, with the average being 0.21 to 0.31 (Fig. 1D). We observed that editing levels in the later fractions (NpA^+^ and CpA^+^) were generally higher than those from fractions of more nascent RNAs (ChA^−^ and ChA^+^). To exclude the possibility that the observed editing change is due to loss of low-level editing sites in introns in later fractions, we carried out the same analysis but excluding intronic editing sites and observed the same pattern of higher editing ratios in the later fractions (Supplemental Fig. S7). Indeed, a “monotonicity *Z*-score” (MZ score) (Wang et al. 2015) calculation showed that the majority of editing sites in the kinetic groups 1 and 2 had positive MZ scores, which reflected an increasing trend in their editing levels across fractions (Fig. 1E). Consistent with this trend, in the CpA^+^ fraction, mostly composed of mature mRNAs, group 1 editing sites had higher final editing levels than the other groups (Fig. 1F). The increasing trend of editing levels of groups 1 and 2 indicates that additional editing may have occurred for these sites as their associated RNA traveled through the nucleoplasm to the cytoplasm. Group 1 sites may have been subjected to the most additional editing, which explains their highest average editing levels in CpA^+^ among all three groups. These observations still hold if higher read coverage thresholds were imposed for the editing sites (Supplemental Fig. S8). Among the known recoding sites in the literature (Pinto et al. 2014), five were identified as editing sites in our data set, four of which were categorized as group 1 events (Supplemental Table S4). All of these recoding sites had positive MZ scores.

**Figure 2.**
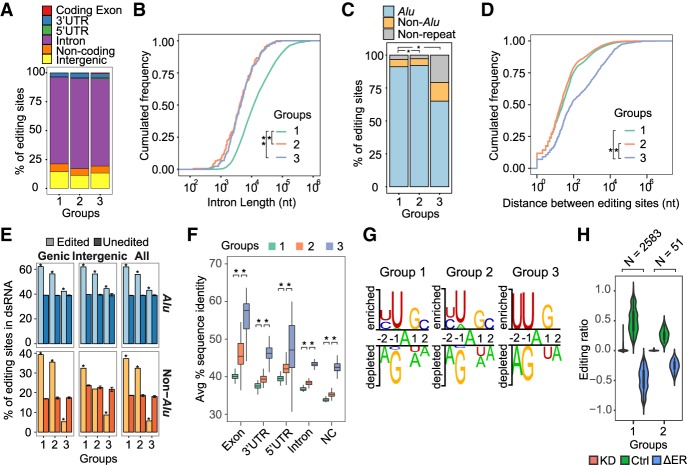
Characteristics of editing sites in different kinetic groups. (*A*) Genomic context of the editing sites. (*B*) Length of introns harboring editing sites: (*) *P* = 1.75 × 10^−4^ for group 1 versus 2; (**) *P* = 3.02 × 10^−4^ for group 1 versus 3 (Kolmogorov–Smirnov test). Groups 2 and 3 were not significantly different. (*C*) Presence of editing sites in *Alu*s, non-*Alu* repeats, and nonrepeats: (*) *P* < 2.2 × 10^−16^ for group 1 versus 3 and group 2 versus 3 (Fisher's exact test). Groups 1 and 2 were not significantly different. (*D*) Closest distance (in nucleotides) between editing sites. For each editing site in a specific group, its closest neighboring editing site (regardless of group) was used to calculate this distance: (*) *P* = <2.2 × 10^−16^ for group 1 versus 3 and group 2 versus 3 (Kolmogorov–Smirnov test). Groups 1 and 2 were not significantly different. (*E*) Percentage of editing sites located in dsRNA structures predicted using BLASTN: (unedited) random As in the same type of genomic region as editing sites (Methods). Error bars represent 95% confidence interval based on 100 random trials; (*) *P* = <2.2 × 10^−16^ (Wilcoxon rank-sum test). (*F*) Average percent sequence identity across primates (Human, Chimp, Gorilla, Orangutan, Rhesus, Baboon, Marmoset, Tarsier, Mouse lemur, Bushbaby, and Tree shrew) calculated using the 46-way MULTIZ alignments (Kent et al. 2002). (NC) noncoding transcripts; (*) *P* < 2.2 × 10^−16^ (Wilcoxon rank-sum test). (*G*) Sequence consensus around the editing sites of each group, with the editing sites at position 0. Sequence motifs were identified via the Two Sample Logo program (Vacic et al. 2006). Random As that were in the same type of genomic region as editing sites were used as negative controls. (*H*) Editing ratios of groups 1 and 2 editing sites in U87MG *ADAR1* knockdown (KD) and control (Ctrl) RNA-seq data (Bahn et al. 2012). The differences in editing ratios (ΔER) were calculated as (KD – Ctrl). The number of editing sites identifiable in the RNA-seq data is shown for each group (*N*).

For all kinetic groups, the majority of editing sites were located in the intronic regions (Fig. 2A). The length of the introns harboring the editing sites of different groups demonstrated significant difference, with introns of group 1 being the longest (Fig. 2B). The same trend was observed by randomly sampling 100 introns from each group for this analysis, excluding the possibility that the sample size difference accounted for the observed trend (Supplemental Fig. S9). Thus, the “earlier” editing sites, those that are edited early in nascent RNA, tend to occur in longer introns.

All kinetic groups were mostly composed of editing sites located in *Alu* regions (Fig. 2C). Notably, group 3 editing sites, those first observed in the NpA^+^ fraction, were more often enriched in nonrepetitive regions than the other two groups. Next, we calculated the distance between two closest editing sites. It should be noted that this distance calculation was not restricted to sites within the same group. The result suggested that groups 1 and 2 editing sites were in tighter clusters of editing compared to group 3 editing sites (Fig. 2D), consistent with the relatively smaller enrichment of group 3 sites in *Alu* regions (Fig. 2C). This observation indicates that early editing sites are more often promiscuously edited than late editing sites. In addition, dsRNA predictions suggested that early editing sites were more enriched in dsRNA regions compared to late editing sites (Fig. 2E). Evolutionary conservation analysis showed that early editing sites resided in relatively less conserved regions (Fig. 2F; Supplemental Methods).

Sequence analysis showed that the three groups were associated with similar sequence biases in the −1 and +1 positions around the editing sites (Fig. 2G), resembling the typical UAG signature of ADAR substrates (Lehmann and Bass 2000). Nonetheless, group 3 editing sites demonstrated additional consensus sequences at the −2 and +2 positions, indicating likely existence of unknown regulatory mechanisms. In an examination of these editing sites upon *ADAR1* knockdown (KD) in U87MG cells (Bahn et al. 2012), we observed that editing in both groups 1 and 2 was lost upon loss of *ADAR1* (Fig. 2H), confirming their dependence on ADAR1. Group 3 was not detected in these data sets possibly due to low editing levels in control cells and inadequate sequencing depth to detect these editing sites.

Together, these analyses support that all editing kinetic groups (1 to 3) consist of ADAR-regulated editing events. Yet, the differences among these groups indicate that additional regulatory mechanisms may exist to impact RNA editing at different stages of RNA maturation, a topic of future investigation.

### The impact of editing on exons with different splicing kinetics

Because our data suggested that most editing sites occurred soon after the RNA was transcribed and likely before some introns were spliced out, we hypothesized that RNA editing affects post-transcriptionally spliced exons more than cotranscriptionally spliced ones. Note that this hypothesis does not preclude the possibility that editing may affect cotranscriptionally spliced exons, depending on the relative kinetics of these two processes on specific exons. To test this hypothesis, we adopted the “completed splicing index” (coSI) metric that quantifies the splicing completeness around an internal exon (Tilgner et al. 2012). We calculated the coSI values to identify cotranscriptionally spliced (coTS) exons and post-transcriptionally spliced (postTS) exons in the U87MG RNA-seq. As in Tilgner et al. (2012), we defined postTS exons as those with coSI ≤ 0.75 in the ChA^+^ fraction and ≥0.95 in the NpA^+^ fraction, and coTS exons as those with coSI ≥ 0.95 in the ChA^−^ fraction. In total, we identified 6104 coTS exons and 3304 postTS exons. We then compared the splicing difference between these two groups of exons upon *ADAR1* KD in U87MG cells. The results showed that the change in exon inclusion in postTS exons was larger than that in coTS exons (Supplemental Fig. S10), suggesting that RNA editing imposes a larger impact on exons undergoing post-transcriptional splicing.

### Editing sites located in the splice site regions often disrupt 3′ splice sites

**Figure 3.**
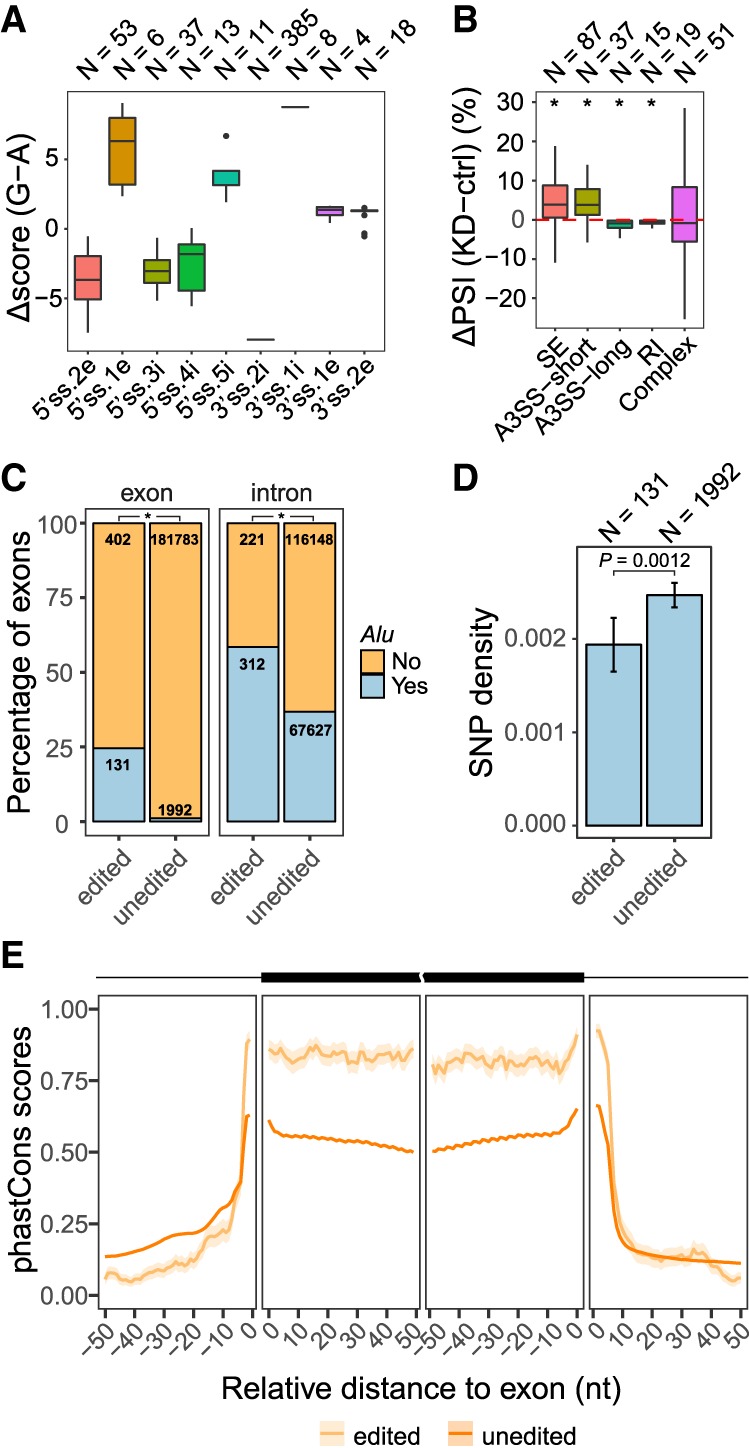
RNA editing sites located in splice site regions. (*A*) Box plot of the difference in splice site strength caused by A-to-I editing. Splice site scores were calculated using the maxEnt method (Yeo and Burge 2004): (5′ss) 5′ splice site; (3′ss) 3′ splice site; (*i*) intron; (*e*) exon; (*N*) number of editing sites. The number before *i* or *e* represents the distance (in nucleotides) from the exon–intron boundaries. Nucleotide positions without any editing sites in our data were excluded from this plot. (*B*) Box plot of PSI changes (ΔPSI) upon *ADAR1* KD in U87MG cells measured via RNA-seq for exons with editing sites at the −2 intronic position of 3′ss (3′ss.2*i*), altering the canonical 3′ acceptor site from AG to GG: (SE) skipped exons; (A3SS-short) alternative 3′ss generating shorter exons given the edited G in the 3′ss; (A3SS-long) alternative 3′ss generating longer exons given the edited G in the 3′ss; (RI) retained introns; (Complex) complex splicing patterns; (*N*) number of exons; (*) *P* < 0.05 for ΔPSI ≠ 0 (Wilcoxon rank-sum test). (*C*) Percentage of exons or their upstream introns that overlap *Alu* repeats: (edited) exons or introns with editing sites in the 3′ss AG sequence; (unedited) all internal exons or introns without editing sites in the 3′ss. The number of exons or introns in each category is shown. The edited groups are significantly more enriched with *Alus* than unedited groups for both panels. (*) *P* < 2.2 × 10^−16^ (Fisher's exact test). (*D*) Density of SNPs (common SNPs in dbSNP version 150) in *Alu*-overlapping exons as described in *C*. Error bars represent standard errors. Edited exons have smaller SNP density than unedited ones (*P* = 0.0012, Wilcoxon rank-sum test). (*E*) Average phastCons scores of non-*Alu*-overlapping skipped exons (described in *C*) and their flanking intronic regions. Exon–intron boundaries are positioned at 0. The shaded area represents standard errors.

Next, we hypothesized that A-to-I editing may affect pre-mRNA splicing by altering splice site sequences. To this end, we searched for editing sites identified in the ChA^−^ fraction that were located in the splice site consensus. Specifically, a 9-mer sequence was defined as the 5′ splice site (5′ss) with 3 nt in the exon and 6 nt in the intron. A 23-mer sequence was defined as the 3′ss with 3 nt in the exon and 20 nt in the intron, based on previous literature (Yeo and Burge 2004). To carry out a comprehensive search, we included both annotated exons (GENCODE v24lift37) and novel exons identified from *ADAR1* KD RNA-seq data sets (Bahn et al. 2012; Methods). A total of 492 editing sites were found to reside in splice site sequences, which were predicted to alter the splice site strength to different degrees (Fig. 3A; Supplemental Table S5; Yeo and Burge 2004). We observed that most (78%, 385) of these editing sites were located in the 3′ss, altering the splice site sequences from AG to GG (3′ss: −2 location). For these sites, A-to-I editing resulted in a dramatic reduction of the predicted splice site strength (Fig. 3A), which is expected since the canonical dinucleotide splice site sequences were altered. We thus focused on this group of exons in the analyses below.

We next examined the splicing patterns of these exons in *ADAR1* KD RNA-seq data (Bahn et al. 2012). A total of 209 such exons were identified with adequate read coverage to calculate their percent spliced-in (PSI) values (Methods). Based on the RNA-seq data, 158 of the 209 exons were categorized into a single category of alternative splicing: skipped exons (SE), alterative 3′ss (A3SS) or retained introns (RI). The remaining exons had complex splicing patterns (e.g., both SE and A3SS). For all exons, we calculated their PSI values in *ADAR1* KD and control data sets. Among the 158 exons with a single alternative splicing type, 137 (87%) had PSI changes in the same direction as expected based on the alteration in the 3′ss sequence by the A-to-G change (Fig. 3B). The expected direction of PSI changes for exons with complex splicing is unknown. It should be noted that the level of PSI change is expected to be low because the editing levels of the 3′ss-altering editing sites are relatively low (mean: 0.42; median: 0.36), and the change from A to I may not completely abolish splicing activity.

As an independent analysis, we searched for splice site–altering editing sites in the NA^−^ samples of ENCODE cell fractionation RNA-seq in HepG2 and K562 cells. Similar to the U87MG data, the majority of splice site–disrupting editing sites are located in the 3′ss and alter AG to GG (Supplemental Fig. S11A), resulting in a total of 498 such unique editing sites if combining data from the three cell lines. To verify their impact on splicing, we analyzed the splicing patterns of the associated exons as described above, using the ENCODE *ADAR1* KD RNA-seq of the respective cell lines (Van Nostrand et al. 2017). Among the 35 exons with a single alternative splicing type, 29 (83%) showed the expected direction of PSI changes upon *ADAR1* KD (Supplemental Fig. S11B). Altogether, our analyses of multiple data sets led to identification of an unexpectedly large number of editing sites located in splice site regions that can potentially alter splicing.

### Exons with A-to-I editing in the 3′ss tend to be highly conserved

To characterize the exons that harbor A-to-I editing sites in their 3′ss, we next examined the overlap of these exons with annotated *Alu* repeats. About 25% of these exons overlapped *Alu* sequences, a much higher percentage than what was observed for control exons that do not have known editing sites in the 3′ss (Fig. 3C). Similarly, the flanking intronic regions of these exons (within 500 nt from the exons) were also enriched with *Alu* repeats (Fig. 3C). This observation is consistent with the fact that A-to-I editing sites often reside in *Alu* elements.

The aforementioned *Alu*-containing exons had editing sites that weaken the 3′ss strength (from AG to GG). This alteration in 3′ss is opposite to the previously reported example, in which A-to-I editing creates a 3′ss (changing AA to AG) and enables *Alu* exonization (Lev-Maor et al. 2007). Thus, it is unlikely that the exons in our study represent *Alu* exonization cases resulting from RNA editing. In contrast, since RNA editing appears to destroy the 3′ss, we asked whether these exons are under relaxed selection pressure for their preservation as exons. Because *Alus* are highly primate-specific, we examined the density of human single-nucleotide polymorphisms (SNPs) in *Alu*-containing exons as a proxy for sequence conservation. We observed that *Alu*-containing exons with 3′ss editing had significantly lower SNP density than control exons (that were also *Alu*-containing, but without known 3′ss editing) (Fig. 3D; Supplemental Methods). The Fay and Wu's H values (Fay and Wu 2000; Pybus et al. 2014) were not significantly different between *Alu*-containing exons with 3′ss editing and the control exons (Supplemental Fig. S12). Thus, it is likely that edited *Alu*-containing exons are under selection to preserve their sequences more than random controls.

Next, we examined the sequence conservation of non-*Alu*-containing exons with 3′ss editing sites across 46 vertebrates spanning primates to fish (Siepel et al. 2005). Since these exons demonstrated multiple types of alternative splicing in the RNA-seq data (Fig. 3B) and because different types of alternative splicing may have distinct conservation patterns, we focused on 96 exons that were alternatively skipped, the largest category of alternative splicing. Compared to control skipped exons from annotation (Katz et al. 2010), the 3′ss-edited exons had significantly higher conserved exonic and splice site sequences (Fig. 3E; Supplemental Methods). Together, both *Alu*-containing and non-*Alu* exons with 3′ss editing showed evidence of higher conservation than their corresponding controls.

Based on Gene Ontology (GO) analysis (Supplemental Methods), the genes that contain 3′ss-edited exons (*Alu* or non-*Alu*) were enriched in functional categories related to protein trafficking, cellular metabolism (protein or energy-related), RNA processing, or cytoskeletal structures (Supplemental Fig. S13). Thus, consistent with the highly conserved nature of the exons, these genes are potentially implicated in critical cellular functions.

### Experimental verification of editing-dependent alternative splicing

**Figure 4.**
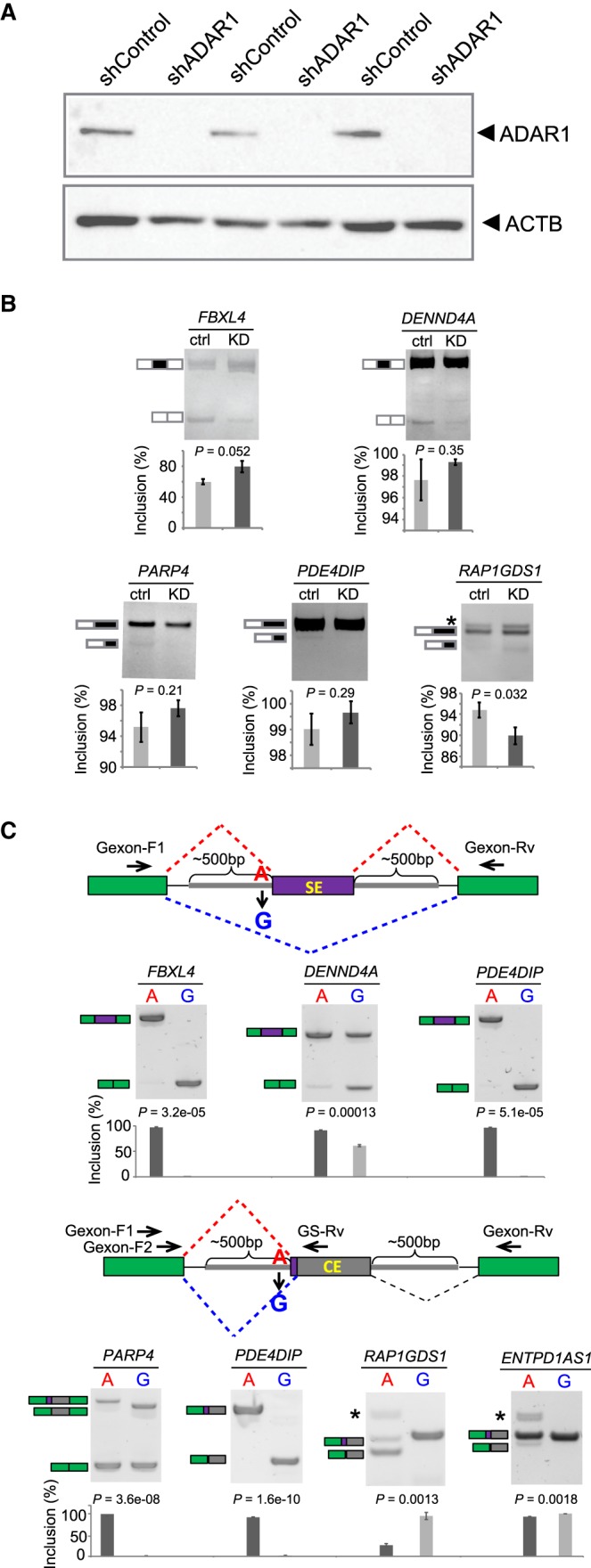
Experimental validation of splicing changes induced by 3′ss editing. (*A*) Western blot of ADAR1 in U87MG cells stably expressing control shRNA (shControl) or *ADAR1* shRNA (shADAR1). Three biological replicates are shown. (*B*) RT-PCR results of endogenous splicing levels of five randomly chosen exons with 3′ss editing. U87MG cells in *A* were used. (Ctrl) shControl; (KD) shADAR1. Exon inclusion levels (%) were calculated based on triplicated experiments. One example gel image is shown. Error bars represent standard deviation. *P*-values were calculated by Student's *t*-test. (*C*) Seven randomly chosen editing-dependent alternative splicing events were validated in minigene systems. The minigene designs to validate exon skipping (*top*) and alternative splice site events (*bottom*) are shown, respectively. Primers used to amplify the splicing products are shown as arrowheads. In the *bottom* diagram, Gexon-F1 and Gexon-Rv represent the forward and reverse primers used for *PARP4*. Gexon-F2 and GS-Rv refer to the forward and gene-specific reverse primers for the other three exons*.* Exon inclusion levels (%) were calculated based on triplicated experiments. One example gel image is shown. Error bars represent standard deviation. *P*-values were calculated by Student's *t*-test.

To experimentally confirm the impact of RNA editing on splicing, we randomly picked five 3′ss-edited exons and tested whether their splicing patterns were altered upon *ADAR1* KD in U87MG cells (Fig. 4A; Supplemental Methods). Note that the endogenous editing levels are generally low, and these five candidates had an average editing level of 0.36. Thus, the endogenous splicing changes of these exons upon *ADAR1* KD are not expected to be high. Nevertheless, all five exons demonstrated splicing changes in the expected direction (although some did not reach statistical significance) (Fig. 4B). Two exons (in genes *FBXL4* and *DENND4A*) were alternatively skipped exons (SEs), with increased exon inclusion levels in *ADAR1* KD cells. This observation is consistent with the expectation that the edited version of the 3′ss (GG) weakens the splicing signal and causes exon skipping. The other three exons each had an alternative 3′ss (A3SS). In these cases, given the editing event, the original 3′ss was partially or completely abolished. Instead, an alternative 3′ss in the downstream (*PARP4*, *PDE4DIP*) or upstream (*RAP1GDS1*) region was used, which created a shorter or longer form of the original exon, respectively.

As a complementary experiment, we performed minigene reporter assays on the aforementioned five exons and two additional examples, all of which had 3′ss editing (Methods; Supplemental Table S6). We created two versions of the constructs for each target, one harboring the unedited (A) and the other for the edited (G) alleles in the 3′ss sequence. For the SE events (in genes *FBXL4*, *DENND4A*, and *PDE4DIP*), the exons had significantly reduced inclusion levels in the G version of the construct (Fig. 4C, top panels). Consistent with the endogenous results described above, these data confirm that RNA editing reduces the 3′ss strength and causes exon skipping. In addition, the four A3SS exons also demonstrated expected splicing differences given the A and G alleles (Fig. 4C, bottom panels), where an alternative 3′ss in the downstream (*PARP4*, *PDE4DIP*) or upstream (*RAP1GDS1*, *ENTPD1AS1*) region was used in the presence of the G allele. Taken together, our experimental results support that RNA editing could alter splicing by modifying the splice site sequences.

### Regulation of splicing by ADAR acting on dsRNA structures

Since the majority of RNA editing sites reside outside of canonical splice sites, we sought to investigate whether RNA editing may affect splicing through other mechanisms. One possible mechanism for these editing sites to modulate splicing is through alteration of splicing regulatory motifs (Fu and Ares 2014). However, a motif analysis similar to that in our previous study (Hsiao et al. 2016a) did not yield significant enrichment of any class of motifs. Hence, we examined a second hypothesis that considers the involvement of RNA secondary structures in splicing regulation.

**Figure 5.**
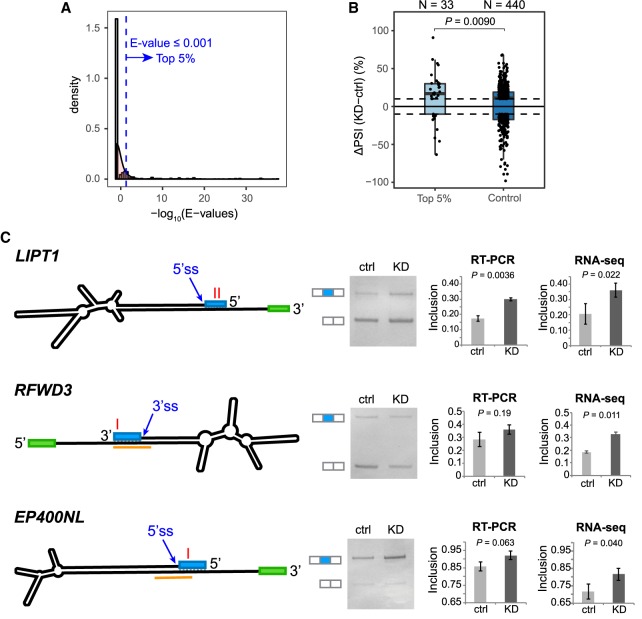
Regulation of splicing by *ADAR* acting on dsRNA structures. (*A*) *E*-value distribution of BLASTN alignments between exon and intron sequences, for exons with ≥10% PSI change upon *ADAR1* KD. A total of 33 exons (top 5% of all) had *E*-value ≤0.001 (blue dashed line). (*B*) Box plot of PSI changes (ΔPSI) upon *ADAR1* KD, for the 33 exons in *A*, and control exons with *E*-value >10 in the BLASTN analysis. Each dot represents an exon. The dashed lines mark ΔPSI = ±10%. The number of exons in each group is shown. *P* = 0.0090 comparing ΔPSI values of the two groups (Wilcoxon rank-sum test). (*C*) Experimental testing of endogenous splicing changes by RT-PCR in *ADAR1* KD and control U87MG cells. The mfold-predicted RNA secondary structures are depicted (not to scale): (blue box) candidate exon under regulation; (green box) a neighboring exon. ADAR1 CLIP peaks are highlighted in orange. The red letter “I” denotes the position of editing sites. Example gel images are shown using RT-PCR products generated by primers on the two flanking exons (white boxes next to the gel images). Mean and SD of exon inclusion levels based on three biological replicates of RT-PCR are shown. Mean and SD of PSI values derived from two biological replicates of RNA-seq are also shown. *P*-values were calculated by Student's *t*-test.

To this end, we first identified a set of exons (GENCODE v24lift37) that showed a PSI change (in either direction) of at least 10% upon *ADAR1* KD in U87MG, HepG2, or K562 cells as reflected in the respective RNA-seq data (Bahn et al. 2012; Van Nostrand et al. 2017). The splicing of these exons could be directly or indirectly affected by ADAR1. To identify direct targets of ADAR1, we further required existence of exonic editing sites or intronic editing sites within 500 nt of the exon boundaries. A total of 555 exons satisfied these criteria. For these exons, we carried out BLASTN analysis to identify those located in dsRNA regions (Altschul et al. 1990) (Methods). Thirty-three exons (27 of which overlapped *Alu* elements) passed a stringent criterion for dsRNA formation (Fig. 5A). Therefore, these exons are likely located in ADAR1-binding substrates, whose splicing is directly affected by ADAR1.

To validate the preceding hypothesis, we examined our previously published CLIP-seq data set (Bahn et al. 2015). Among the 33 exons, 82% were located in dsRNA structures that were associated with ADAR1 CLIP peaks, providing strong support for ADAR1 binding. Furthermore, since the initial requirement of exon PSI change (≥10%) upon *ADAR1* KD was not direction-specific, we asked whether the 33 predicted ADAR1 direct targets demonstrated splicing change in a particular direction. The data showed that the majority (73%) of the 33 exons had increased PSI values upon *ADAR1* KD (Fig. 5B). These data suggest that ADAR1 represses splicing of these exons that reside in dsRNA regions in the pre-mRNA.

To provide experimental validation of the ADAR1-dependent splicing changes, we tested the splicing patterns of four exons in U87MG cells of *ADAR1* KD. These exons were randomly chosen from those predicted to be repressed by ADAR1 and associated with long dsRNA structures in the preceding analyses, the structures of which were also confirmed by mfold (Supplemental Fig. S14; Zuker et al. 1999). Three of the four exons had CLIP-seq binding sites close to the associated exon–intron boundaries (Fig. 5C; Supplemental Fig. S14). Endogenous splicing inclusion levels of these exons were estimated via RT-PCR followed by gel electrophoresis. Three exons demonstrated a trend of higher splicing levels upon *ADAR1* KD, consistent with the predictions via RNA-seq (Fig. 5C). Note that the magnitudes of these splicing changes are generally small, which may explain the lack of statistical significance for two of the three exons. The fourth exon (in the gene *C17orf67*) failed this experiment due to existence of multiple PCR amplification products indicating likely unspecific primers or complex splicing patterns (Supplemental Fig. S15).

These results suggest that ADAR1 may bind to dsRNA structures and repress splicing of the neighboring exons. Since we have shown that RNA editing occurs early in the nascent pre-mRNA, and it is known that splicing is also cotranscriptional (Bhatt et al. 2012), this observation likely reflects the competition between ADAR1 and the spliceosomal complex to bind to and/or process the pre-mRNA.

## Discussion

RNA editing and splicing are both RNA processing steps that may significantly diversify gene expression. An increasing number of studies have examined the intricate crosstalk between these processes (Solomon et al. 2013; Mazloomian and Meyer 2015; Licht et al. 2016). In general, there are two broad categories of mechanisms via which RNA editing may influence alternative splicing. In the first category, the RNA editing sites themselves impact splicing directly, by altering *cis*-regulatory sequences or by changing the stability of dsRNA structures, which may affect the interaction of splicing machineries with RNA. In the second category, the editing machinery (ADAR proteins), instead of editing sites, influences splicing either by competitively binding to dsRNA regions and precluding access of splicing machineries to the RNA or by recruiting *trans*-acting factors that regulate splicing.

The impact of RNA editing on splicing has been demonstrated in a number of example genes, although the underlying mechanisms sometimes remain unknown (Higuchi et al. 2000; Agrawal and Stormo 2005; Solomon et al. 2013; St Laurent et al. 2013; Goldberg et al. 2017). Despite the increasing recognition of the complex interplay between various post-transcriptional gene regulation steps, a global understanding of the influence of RNA editing on splicing is still lacking. Here, we fill in this gap by performing a genome-wide study that leverages subcellular fractionation RNA-seq data. We first showed that the vast majority of RNA editing occurs cotranscriptionally prior to polyadenylation in human cells. This observation provided a foundation for the hypothesis that many RNA editing events (i.e., the resulted editing sites or the ADAR binding events) may alter splicing. Indeed, using a combination of different types of RNA-seq data sets, we identified more than 500 editing events that could alter splicing by changing splice site sequences or through ADAR's interaction with dsRNA structures.

The usage of chromatin-associated nonpolyadenylated (i.e., ChA^−^) RNA-seq is essential to this study. In identifying RNA editing sites using standard polyadenylated RNA-seq data, it is a general practice to exclude RNA–DNA mismatches located in the splice site regions from the list of predicted editing sites (Lee et al. 2013). This practice is due to the concern that these sites are likely false positives resulting from reads erroneously mapped to the exon–intron boundaries because standard RNA-seq should be depleted of such “unspliced” reads. Thus, previous studies likely missed many RNA editing sites in the splice site regions. In contrast, in ChA^−^ RNA, intronic reads are very abundant (Supplemental Fig. S2), reflecting incomplete splicing (Bhatt et al. 2012). Thus, these data afforded a unique opportunity to capture RNA editing sites in the 5′ss and 3′ss sequences.

In this study, we identified exons whose splicing was likely affected by ADAR1's binding to dsRNA structures. These exons, 33 in total, were required to form stable dsRNA structures with their neighboring intronic regions. The exons were also required to demonstrate at least 10% change in PSI values upon *ADAR1* KD, in either direction. Nonetheless, we observed that the majority of these exons had positive PSI changes upon *ADAR1* KD, suggesting that ADAR1 plays a repressive role in their splicing. It should be noted that it is unlikely that the editing sites themselves alter the secondary structures, since the predicted free energy of these structures barely changed when As were substituted by Gs. Thus, the most likely model to explain these data is that binding of ADAR1 to dsRNA prevented the spliceosome or splicing regulators from accessing the RNA or enabling splicing of the related exons. Similar examples were reported in the literature (Reenan et al. 2000; Rieder et al. 2013), and our study expanded the repertoire of exons regulated by this mechanism.

It should be noted that the endogenous splicing changes imposed by editing or ADAR1 binding to dsRNAs are generally small, which is likely due to the relatively low level of RNA editing for most sites. We applied a series of stringent criteria to identify exons whose splicing is likely affected by ADAR1 binding. The small number of exons that resulted from this analysis represent highly confident predictions, which may constitute only a fraction of all exons influenced by this mechanism. Although the splicing change of each exon is small, the collective changes exerted by many events together may confer substantial impacts on cellular function. In addition, such functional impact of RNA editing may be highly important under certain conditions, such as environmental stress or diseases, a subject for future investigations.

Lastly, we found that exons with editing sites in the 3′ss AG sequences were highly conserved compared to controls. This observation indicates that these exons and their host genes may have important functional roles. In addition, RNA editing, by weakening a canonical 3′ss, introduces novel splicing patterns to these exons. These novel gene expression products, although often having low endogenous levels, may diversify the transcriptome and provide evolutionary substrates for selection without perturbing cellular function significantly.

## Methods

### Cell fractionation and RNA-seq library construction

U87MG cells were fractionated as previously described (Bhatt et al. 2012) with some modifications. Briefly, 5 × 10^6^ U87MG cells were treated with the plasma membrane lysis buffer (10 mM Tris-HCl [pH 7.5], 0.1%–0.15% Nonidet P-40, 150 mM NaCl) on ice for 4 min after homogenization by flicking. The sample was centrifuged for 10 min at 4°C, 15,000*g*, after loading the lysate onto 24% sucrose cushion (24% RNase-free sucrose in plasma membrane lysis buffer) using large orifice tips. The supernatant was extracted as the cytoplasmic fraction. After washing with 1× PBS/1 mM EDTA, the pellet was resuspended in 100 µL of prechilled glycerol buffer (20 mM Tris-HCl [pH 7.9], 75 mM NaCl, 0.5 mM EDTA, 0.85 mM DTT, 0.125 mM PMSF, 50% glycerol) by gentle flicking of the tube. An equal volume (100 µL) of nuclei lysis buffer (10 mM HEPES [pH 7.6], 1 mM DTT, 7.5 mM MgCl_2_, 0.2 mM EDTA, 0.3 M NaCl, 1 M Urea, 1% Nonidet P-40) was added. The sample was then vortexed vigorously (highest setting on vortex) for 2 × 2 seconds. After incubation for 10 min on ice, the nucleoplasm and chromatin fraction were then separated by centrifugation at 4°C, 15,000*g*, for 2 min. Fractionation efficiency was validated by Western blotting using antibodies specific to marker proteins for each fraction: β-tubulin (Sigma, Cat#T8328) for cytoplasm, U1-70k (a kind gift from Dr. Douglas Black) for nucleoplasm, and Histone 3 (Abcam, Cat#ab1791) for chromatin.

Total RNA was extracted from each fractionated sample using TRIzol LS (Thermo Fisher Scientific, Cat# 10296028), and ribosomal RNA was depleted using the RiboMinus Transcriptome Isolation Kit (Thermo Fisher Scientific, Cat# K1550-02) according to the manufacturer's instructions. Polyadenylated RNA was then selected by Oligo (dT)_25_ Dynabeads (Thermo Fisher Scientific, Cat# 61002) following the instructions provided by manufacturer. The flow-through was collected, and the RNA was extracted using TRIzol LS as nonpolyadenylated RNA. Three biological replicates (independent cell culture) were obtained for each fraction.

To prepare for RNA-seq libraries, we started with 50 ng RNA extracted from each sample. Paired-end strand-specific RNA-seq was performed with the NEBNext Ultra Directional RNA Library Prep Kit (NEB, Cat# E7420) with the following adjustments to the manufacturer's instructions: Fragmentation was performed for 12 min at 94°C, and PCR cycling was set to 12 cycles. Unique index was ligated to each sample RNA library construct using the NEBNext Multiplex Oligos for Illumina Index Primers Sets 1 and 2 (NEB, Cat# E7335S & E7500S).

### Detection of RNA editing sites

The RNA-seq data sets were mapped to the hg19 genome and Ensembl (Release 75) transcriptome (Yates et al. 2016) using a stringent mapping method (named RASER) described in our previous work (Ahn and Xiao 2015). RASER parameters were specified as m = 0.05 and b = 0.03. Uniquely mapped reads were retained for further analyses. We realigned a subset of reads to the GRCh38 (hg38) assembly to test whether the version of human genome assembly will lead to a significant difference in the alignment results. We observed highly similar results using hg19 and hg38-based alignments, with an overlap rate of >96%, suggesting that the version of genome assembly does not affect our conclusions significantly. To capture hyperedited reads, we took an approach similar to that described in Porath et al. (2014). Specifically, we collected all unmapped read pairs and converted all As to Gs in the reads, thus creating read sequences with C, G, and T nucleotides only. Likewise, we created a 3-nt version of the hg19 genome consisting of only C, G, and T bases. The converted reads were aligned against the converted hg19 genome, and uniquely aligned read pairs were retained. After mapping, the original A nucleotides in the reads were reinstated (Supplemental Material). Reads mapped in the hyperediting alignment step were pooled with those uniquely mapped in the first round of alignment. Triplicates of the same experiment were combined for RNA editing detection to increase read coverage. Mismatches identified in the final mapped reads were examined. Final RNA editing sites were identified after removing likely false positives due to sequencing errors, inaccurate mapping to repetitive sequences or spliced junctions, or technical biases (such as strand bias of reads) (Bahn et al. 2012; Lee et al. 2013; Zhang and Xiao 2015). In addition, a final predicted editing site was required to be supported by at least two reads with the edited G nucleotide, and covered by at least five reads in total. It should be noted that predicted editing sites located close to exon–intron junctions were not excluded from those identified in the ChA^−^ fraction but were excluded from the list of editing sites identified in the other fractions.

### Definition of RNA editing kinetic groups

We used ChA^−^, ChA^+^, NpA^+^, and CpA^+^ samples to represent the approximate kinetic order of RNA maturation. RNA editing sites identified from all fractions were pooled together to obtain the overall set of editing sites considered in this study. For each editing site, we then examined whether it was edited, unedited, or undetermined in each fraction as follows: (1) A site was deemed “edited” in one fraction if it had ≥1 read with the edited G nucleotide, regardless of the total number of reads; (2) a site was deemed “unedited” in one fraction if its total read coverage was at least *k* (see below), but no read contained the edited G; or (3) a site was deemed “undetermined” if its read coverage was inadequate (<*k*), and no read contained the edited G. Given these three possibilities, an editing site may fall into one of 65 possible categories, considering its status in the four fractions (Supplemental Table S3). In this study, we focused on the categories that have unambiguous interpretation regarding the kinetic timing of an editing site's occurrence and further organized them into four groups (Fig. 1A).

To unambiguously determine whether an editing site was “unedited” in a specific fraction, the parameter *k* was set to be 20 in order to achieve adequate statistical power to capture edited reads (Li et al. 2012). One exception was the *k*-value for group 1 editing sites. A group 1 editing site was required to be “edited” in ChA^−^, and either “edited” or “undetermined” in the other three fractions (Fig. 1A). For this group, we used *k* = 5, a relatively low value that increases the chance of calling “unedited” sites in the other fractions and being excluded from group 1. This cutoff thus results in a conservative estimate of the number of group 1 editing site. For example, if *k* was set to be 10 or 20, a total of 77,169 and 81,248 group 1 editing sites were identified, respectively, a much larger number than the 68,539 sites resulted from *k* = 5. Thus, the estimated proportion of group 1 (cotranscriptional) editing sites in this study is relatively conservative.

From the ENCODE Project Consortium, we obtained RNA-seq data of subcellular fractions of HepG2 and K562 cells. Each cell line had data sets derived from nuclear poly(A)^−^ (NA^−^), nuclear poly(A)^+^ (NA^+^), and cytoplasmic poly(A)^+^ (CA^+^) RNA (Djebali et al. 2012). Using these data, the editing kinetic groups of A-to-I editing sites were defined similarly as for the U87MG data. Specifically, group 1 editing sites included those edited in NA^−^, and either edited or undetermined in NA^+^ and CA^+^. Group 2 editing sites included those that were unedited in NA^−^, but edited in NA^+^, and either edited or undetermined in CA^+^. Group 3 consisted of sites that were unedited in neither NA^−^ nor NA^+^, but edited in CA^+^. For groups 1, 2, and 3, the *k* parameters were set to be 5, 20, and 20, respectively, in a similar way as the U87MG data.

The editing kinetic groups of A-to-I editing sites in mouse dendritic cells were derived from published time-course 4sU-labeled RNA-seq data sets (Rabani et al. 2014). 4sU was used to label newly synthesized RNA at different time points following LPS stimulation. RNA editing sites in 4sU-labeled RNA were compared to those derived from total RNA collected at 180 minutes after LPS stimulation (Total-180 min), which was the last time point available in this data set. Early edited sites were defined as those with editing ratio (ER) > 0 in both 4sU-RNA and Total-180 min samples, whose ER did not change significantly between these two samples (Fisher's exact test, *P* > 0.05). Intermediate group consisted of editing sites with ER > 0 in both 4sU-RNA and Total-180 min, but significant increase of ER was observed in Total-180 min relative to 4sU-RNA (Fisher's exact test, *P* < 0.05). Late editing refers to those editing sites with ER = 0 in 4sU-RNA, and ER > 0 in Total-180 min RNA.

### Prediction of novel exons and PSI calculation

We used our previously developed method (Lee et al. 2011) and the GENCODE v24lift37 transcriptome (Harrow et al. 2012) as a reference annotation to identify novel exons in RNA-seq data of *ADAR1* KD and control samples. This method examines spliced junction reads and read coverage in putative exonic and intronic regions to call exons. These putative exons were compared with those in the reference annotation to identify novel exons. We required a novel exon to have a read density of ≥10 (normalized by read length and exon length) and ≥2 exon junction reads supporting each exon boundary. The PSI values of annotated and novel exons were calculated following a previously developed protocol (Schafer et al. 2015). Each exon was required to have ≥10 reads in total (corresponding to exon inclusion and exclusion) or ≥2 exon exclusion reads in the KD or control data set.

### Prediction of RNA secondary structures

To test whether the predicted A-to-I editing sites are located in or near dsRNA regions (Fig. 2E), we used a previously published approach (Bahn et al. 2012). We extracted 4001-bp genomic sequences with the editing sites located in the middle. This sequence was then reverse-complemented and aligned against the immediate neighborhood (200 bp flanking each side) of the editing sites using BLASTN (Altschul et al. 1990). Excluding the case of self-alignment, if the second-best alignment has an alignment length ≥50 and total identity ≥80% (parameters chosen to include most known A-I editing with strong dsRNA structures), we concluded that the editing site resides in dsRNA structures.

For the structure analysis presented in Figure 5A, we collected a list of exons with at least 10% PSI changes upon *ADAR1* KD (henceforth called “AS exons”) based on the RNA-seq data of three human cell lines (U87MG, K562, and HepG2). We focused on the AS exons with exonic or intronic editing sites within 500 nt. AS exons without editing sites in these regions were used as controls. To identify candidate dsRNA structures encompassing the AS exons, we ran BLASTN (Altschul et al. 1990) to align the AS exonic sequences and the reverse-complement sequences of the corresponding flanking introns. Structure with a percent identity >70% and alignment *E*-values ≤0.001 were retained as putative dsRNA structures associated with the AS exons.

### Minigene constructs

Genomic regions encompassing the candidate exon and ∼500 nt upstream and downstream flanking introns (including the editing site) were amplified using genomic DNA from HEK293 cells. Primers used in this study are listed in Supplemental Table S6. After double digestion by HindIII and SacII or EcoRI and SacII, the DNA fragment was subcloned into a splicing reporter used in a previous study (Xiao et al. 2009).

## Data access

RNA-seq data derived from subcellular fractions of the U87MG cells from this study have been submitted to the NCBI Gene Expression Omnibus (GEO; https://www.ncbi.nlm.nih.gov/geo/) under accession number GSE105773. Custom scripts are available in Supplemental_Code.zip.

## Supplementary Material

Supplemental Material
