# Supplementary material for: RNA editing in nascent RNA affects pre-mRNA splicing
Source: Genome Res. 2018 Jun;28(6):812–23. doi: 10.1101/gr.231209.117 (PMC5991522; doi:10.1101/gr.231209.117)
Supplement: Supplemental Material [file supp_28_6_812__index.html]

RNA editing in nascent RNA affects pre-mRNA splicing — Supplemental Material 

# RNA editing in nascent RNA affects pre-mRNA splicing

## Supplemental Material

- Supplemental\_Fig\_S1.pdf
- Supplemental\_Fig\_S2.pdf
- Supplemental\_Fig\_S3.pdf
- Supplemental\_Fig\_S4.pdf
- Supplemental\_Fig\_S5.pdf
- Supplemental\_Fig\_S6.pdf
- Supplemental\_Fig\_S7.pdf
- Supplemental\_Fig\_S8.pdf
- Supplemental\_Fig\_S9.pdf
- Supplemental\_Fig\_S10.pdf
- Supplemental\_Fig\_S11.pdf
- Supplemental\_Fig\_S12.pdf
- Supplemental\_Fig\_S13.pdf
- Supplemental\_Fig\_S14.pdf
- Supplemental\_Fig\_S15.pdf
- Supplemental\_Code.zip
- Supplemental\_Methods.pdf
- Supplemental\_Table\_S1.xlsx
- Supplemental\_Table\_S2.xlsx
- Supplemental\_Table\_S3.xlsx
- Supplemental\_Table\_S4.xlsx
- Supplemental\_Table\_S5.xlsx
- Supplemental\_Table\_S6.xlsx
