# Supplementary material for: RNA editing in nascent RNA affects pre-mRNA splicing
Source: Genome Res. 2018 Jun;28(6):812–23. doi: 10.1101/gr.231209.117 (PMC5991522; doi:10.1101/gr.231209.117)
Supplement: Supplemental Material [file supp_gr.231209.117_Supplemental_Fig_S1.pdf]

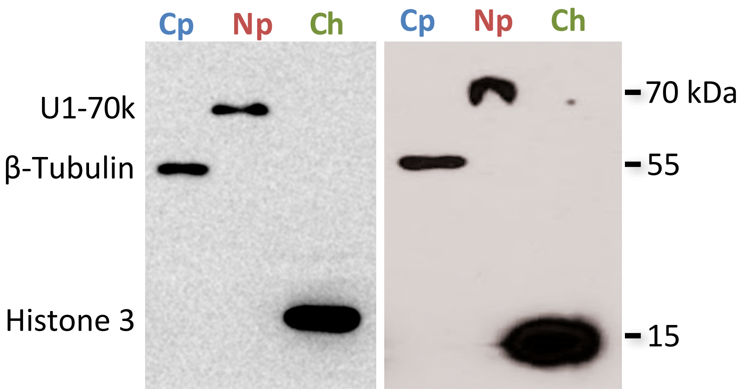

Supplemental Fig S1. U87MG subcellular fractionation validated by Western blotting using the antibody specific to the marker protein of each fraction: β-tubulin for cytoplasm (Cp), U1-70k for nucleoplasm (Np), and Histone 3 for chromatin (Ch).
