# Supplementary material for: RNA editing in nascent RNA affects pre-mRNA splicing
Source: Genome Res. 2018 Jun;28(6):812–23. doi: 10.1101/gr.231209.117 (PMC5991522; doi:10.1101/gr.231209.117)
Supplement: Supplemental Material [file supp_gr.231209.117_Supplemental_Fig_S2.pdf]

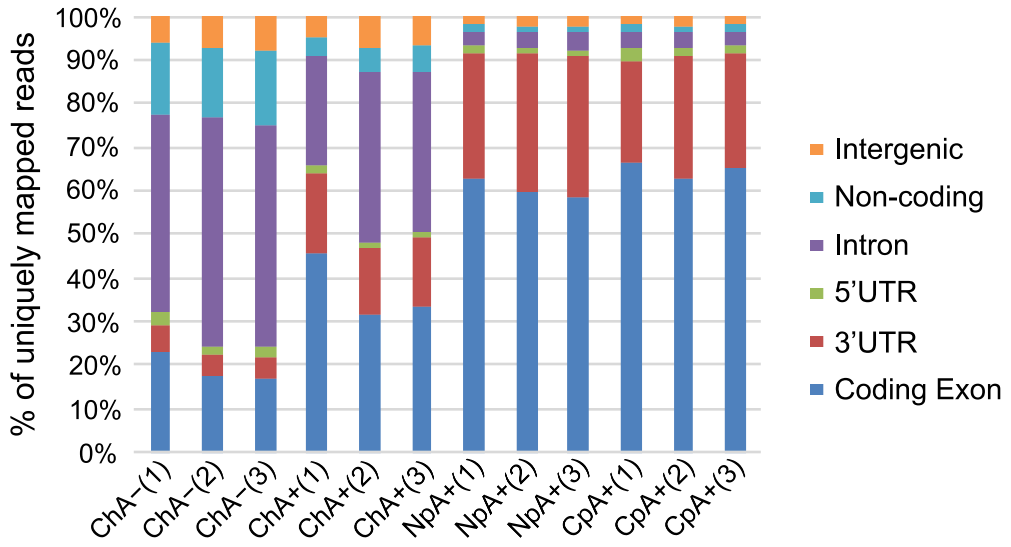

Supplemental Fig S2. Genomic context of uniquely mapped reads. Percentage of uniquely mapped reads for different genomic regions are shown for triplicates of each subcellular fraction. Replicates are named as, for example, ChA−(1), ChA−(2), ChA−(3).
