# Supplementary material for: RNA editing in nascent RNA affects pre-mRNA splicing
Source: Genome Res. 2018 Jun;28(6):812–23. doi: 10.1101/gr.231209.117 (PMC5991522; doi:10.1101/gr.231209.117)
Supplement: Supplemental Material [file supp_gr.231209.117_Supplemental_Fig_S3.pdf]

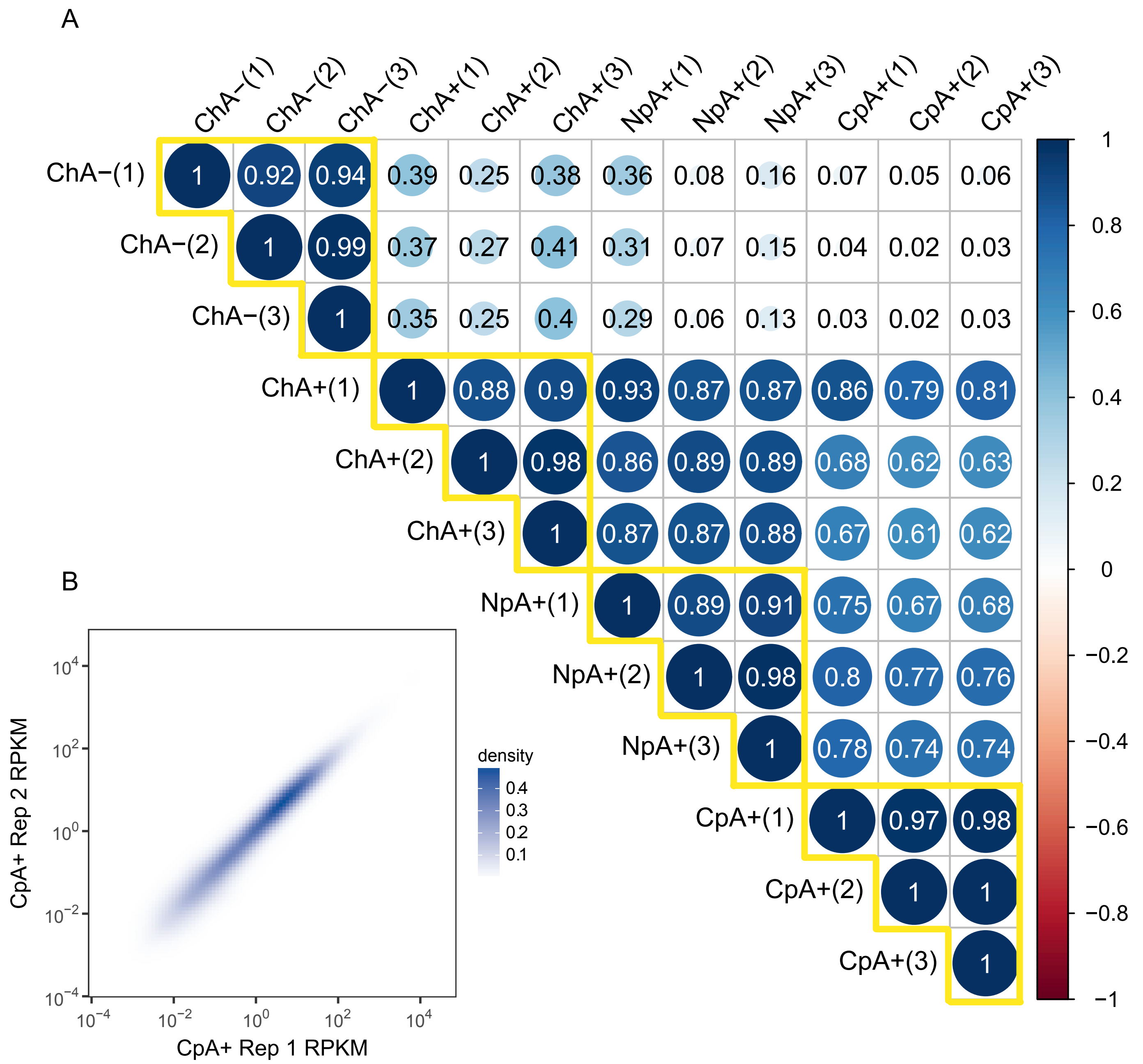

Supplemental Fig S3. Comparison of the three replicates of each cell fraction. (A) Correlation cofficients of RPKM values between pairs of samples. Replicates are named as, for example, ChA-(1), ChA-(2) and ChA-(3). Replicates of the same cell fraction are highlighted by yellow boxes. (B) An example correlation plot of RPKM values (two replicates of CpA+ data). A density plot is shown to summarize the overlapping data points in tight clusters.
