# Supplementary material for: RNA editing in nascent RNA affects pre-mRNA splicing
Source: Genome Res. 2018 Jun;28(6):812–23. doi: 10.1101/gr.231209.117 (PMC5991522; doi:10.1101/gr.231209.117)
Supplement: Supplemental Material [file supp_gr.231209.117_Supplemental_Fig_S4.pdf]

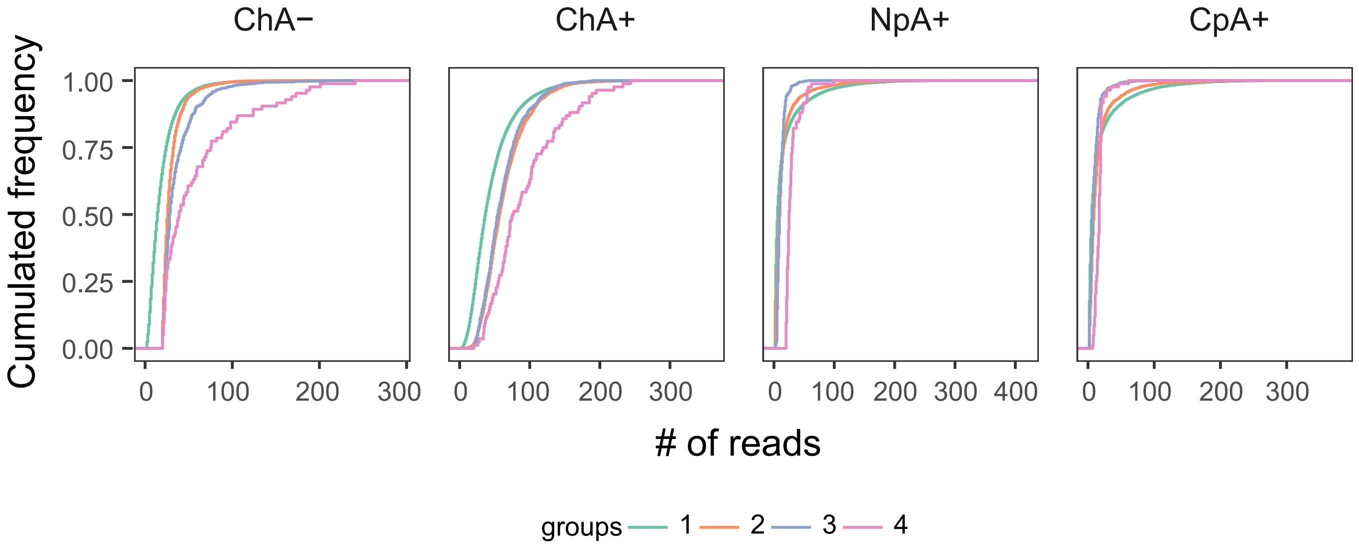

Supplemental Fig S4. Read coverage in each subcellular fraction for editing sites in the 4 kinetic groups. Group 4 editing sites showed higher coverage in ChA- and ChA+ fractions than editing sites in other groups, which suggests that the absence of edited reads for group 4 editing sites in the chromatin fractions was not due to lack of detection power. Similarly, editing sites in groups 2 and 3 also had higher coverage in ChA- and ChA+ fractions than those in group 1. The data suggest that our definition for editing kinetics groups are not biased by read coverage.
