# Supplementary material for: RNA editing in nascent RNA affects pre-mRNA splicing
Source: Genome Res. 2018 Jun;28(6):812–23. doi: 10.1101/gr.231209.117 (PMC5991522; doi:10.1101/gr.231209.117)
Supplement: Supplemental Material [file supp_gr.231209.117_Supplemental_Fig_S5.pdf]

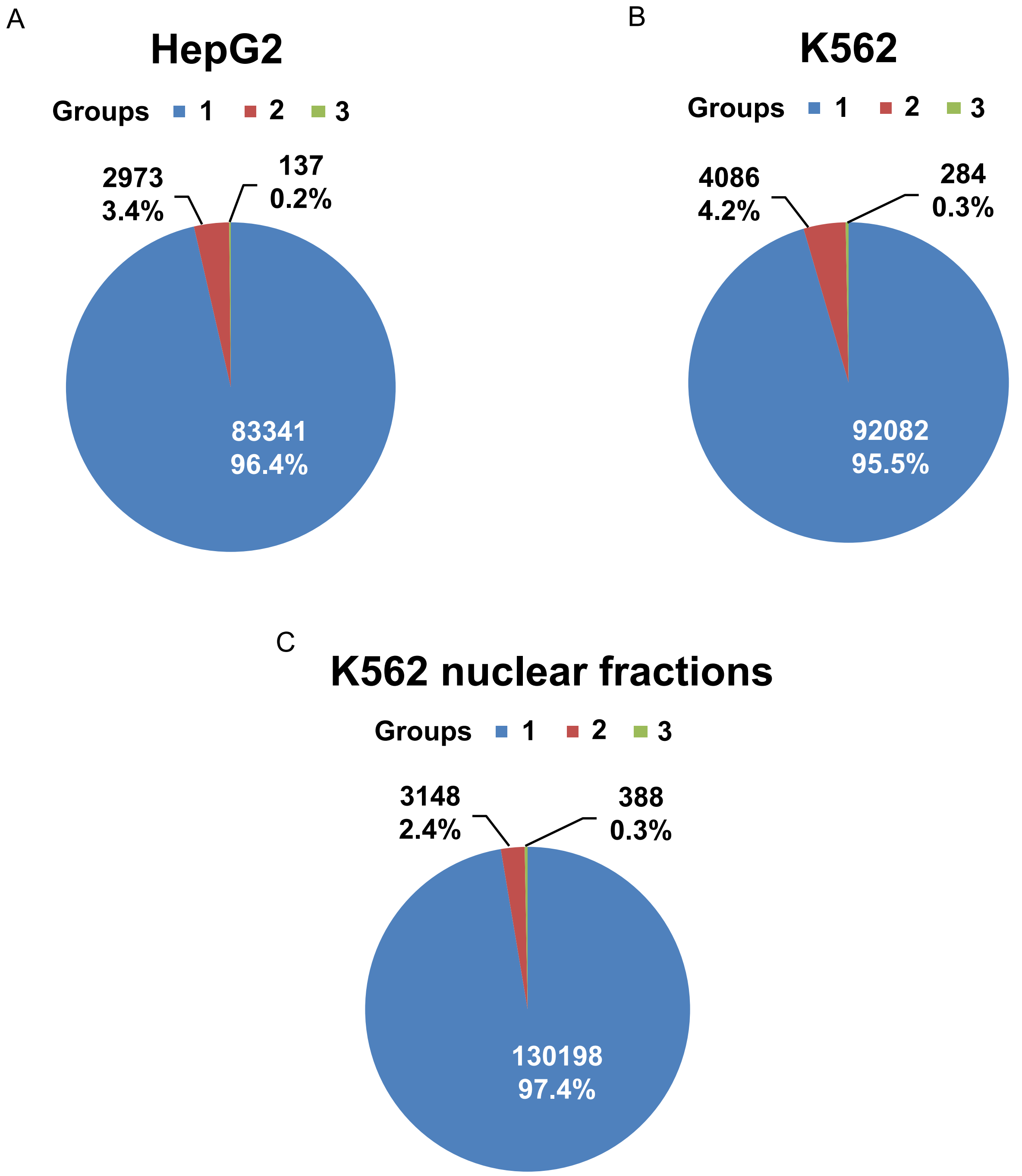

Supplemental Fig S5. Editing kinetic groups of A-to-I editing sites in (A) HepG2 and (B) K562 cells, obtained using cellular fractionation NA-, NA+, and CA+ RNA-seq data by the ENCODE consortium. See Methods for defintion of groups. (C) Editing kinetic groups of A-to-I editing sites in K562 cells, obtained using nuclear fractionations (chromatin and nucleoplasmic) and CA+ RNA-seq data by the ENCODE consortium. Grouping was defined similarly as for U87MG data.
