# Supplementary material for: RNA editing in nascent RNA affects pre-mRNA splicing
Source: Genome Res. 2018 Jun;28(6):812–23. doi: 10.1101/gr.231209.117 (PMC5991522; doi:10.1101/gr.231209.117)
Supplement: Supplemental Material [file supp_gr.231209.117_Supplemental_Fig_S6.pdf]

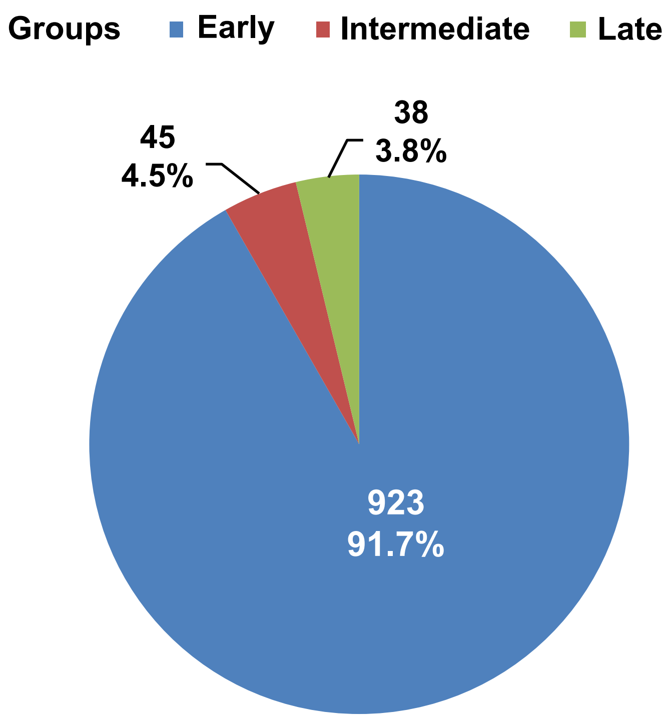

Supplemental Fig S6. Editing kinetic groups of A-to-I editing sites in mouse dendritic cells, obtained using time-course 4sU-labeled RNA-Seq data sets. See Methods for details.
