# Supplementary material for: RNA editing in nascent RNA affects pre-mRNA splicing
Source: Genome Res. 2018 Jun;28(6):812–23. doi: 10.1101/gr.231209.117 (PMC5991522; doi:10.1101/gr.231209.117)
Supplement: Supplemental Material [file supp_gr.231209.117_Supplemental_Fig_S7.pdf]

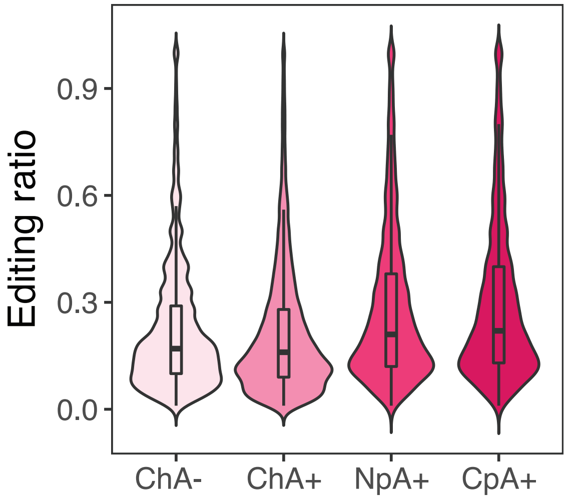

Supplemental Fig S7. Related to Fig. 1D. Editing ratio distribution of the editing sites in Fig. 1C (union of all four kinetic groups) in each subcellular fraction, excluding those that were in the introns.
