# Supplementary material for: RNA editing in nascent RNA affects pre-mRNA splicing
Source: Genome Res. 2018 Jun;28(6):812–23. doi: 10.1101/gr.231209.117 (PMC5991522; doi:10.1101/gr.231209.117)
Supplement: Supplemental Material [file supp_gr.231209.117_Supplemental_Fig_S8.pdf]

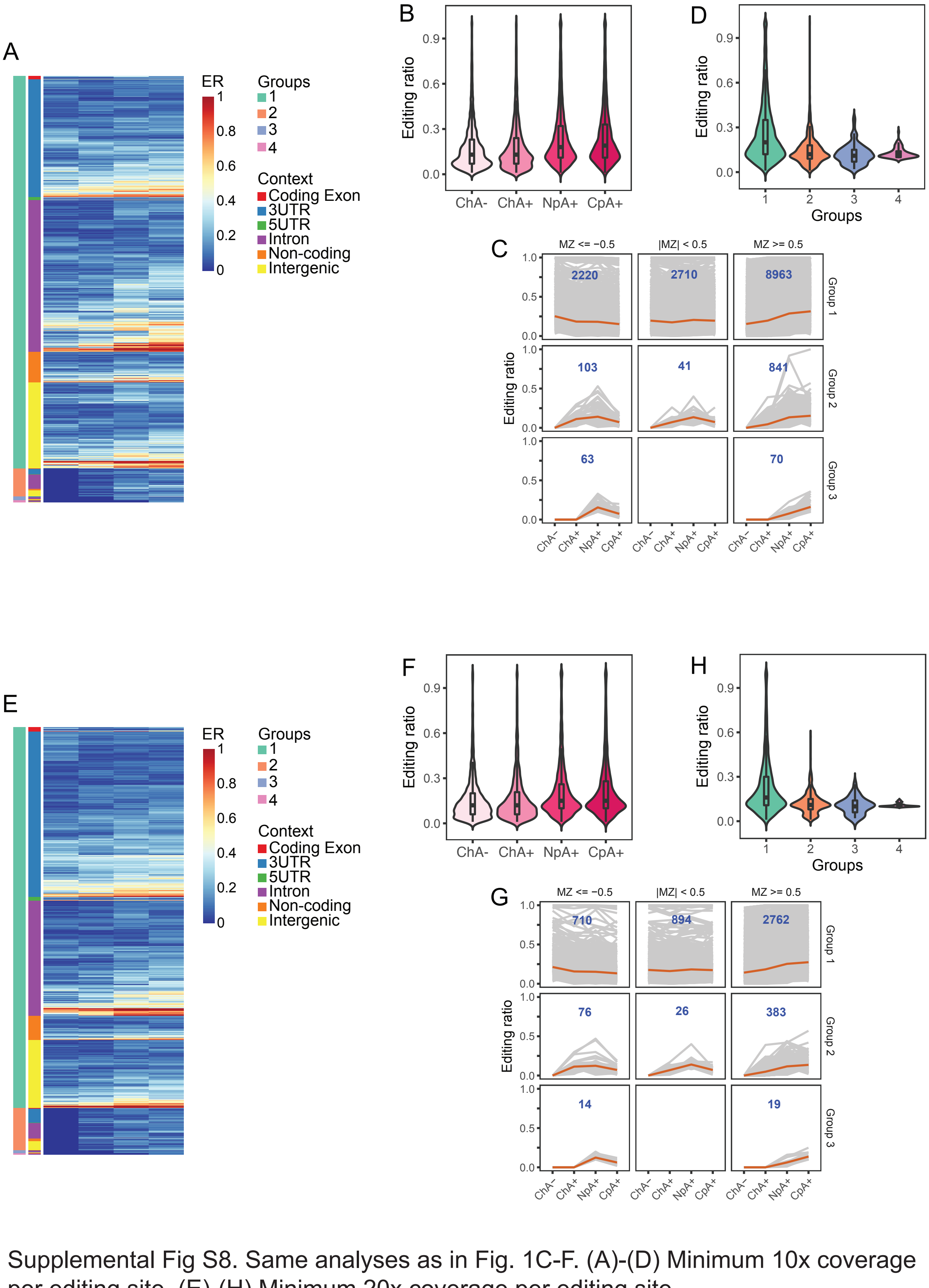

Supplemental Fig S8. Same analyses as in Fig. 1C-F. (A)-(D) Minimum 10x coverage per editing site. (E)-(H) Minimum 20x coverage per editing site.
