# Supplementary material for: RNA editing in nascent RNA affects pre-mRNA splicing
Source: Genome Res. 2018 Jun;28(6):812–23. doi: 10.1101/gr.231209.117 (PMC5991522; doi:10.1101/gr.231209.117)
Supplement: Supplemental Material [file supp_gr.231209.117_Supplemental_Fig_S9.pdf]

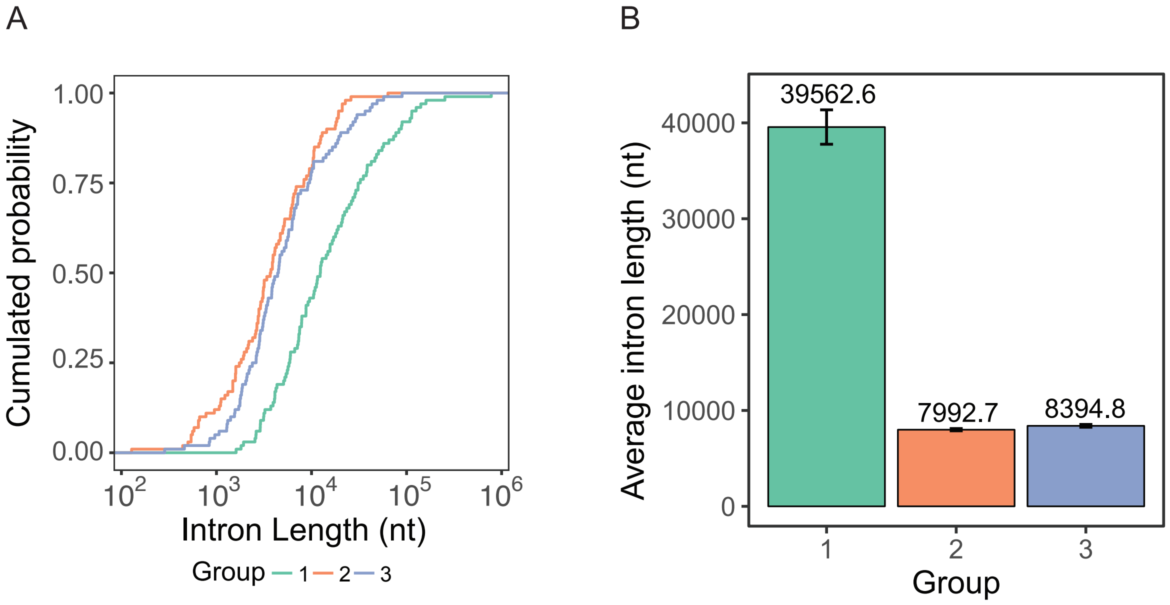


Supplemental Fig S9. (A) Intron length distriubtion of an example random trial that sampled 100 random edited introns from each group. (B) Average intron length in each editing kinetic group over 100 random trials. The error bars showed 95% confidence intervals, and the numbers above each bar represent the average length per group.
