# Supplementary material for: RNA editing in nascent RNA affects pre-mRNA splicing
Source: Genome Res. 2018 Jun;28(6):812–23. doi: 10.1101/gr.231209.117 (PMC5991522; doi:10.1101/gr.231209.117)
Supplement: Supplemental Material [file supp_gr.231209.117_Supplemental_Fig_S10.pdf]

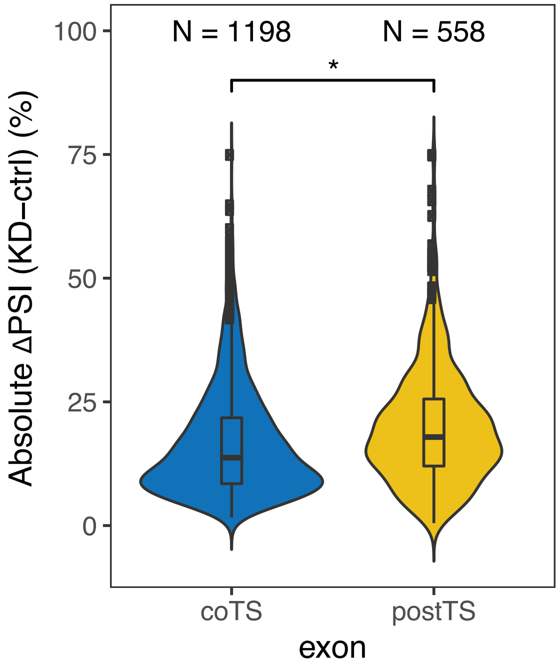

Supplemental Fig S10. Absolute change in PSI of coTS exons vs. postTS exons identified in U87MG cells. N: Number of exons. * P = 3.5e-11, Wilcoxon rank sum test.
