# Supplementary material for: RNA editing in nascent RNA affects pre-mRNA splicing
Source: Genome Res. 2018 Jun;28(6):812–23. doi: 10.1101/gr.231209.117 (PMC5991522; doi:10.1101/gr.231209.117)
Supplement: Supplemental Material [file supp_gr.231209.117_Supplemental_Fig_S11.pdf]

A

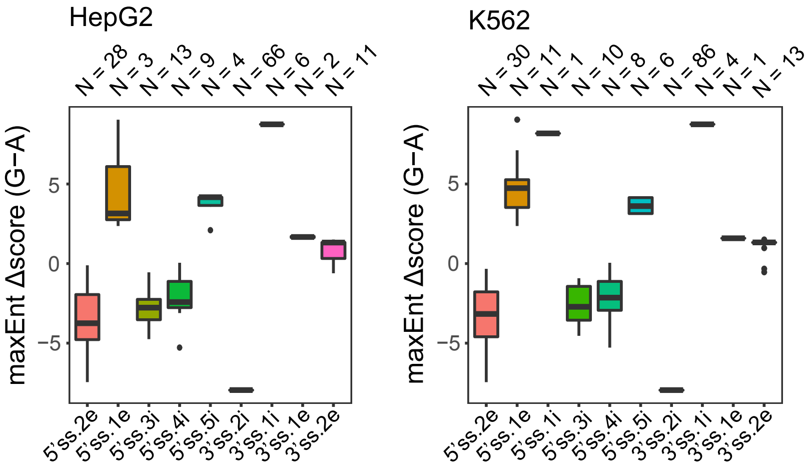

B

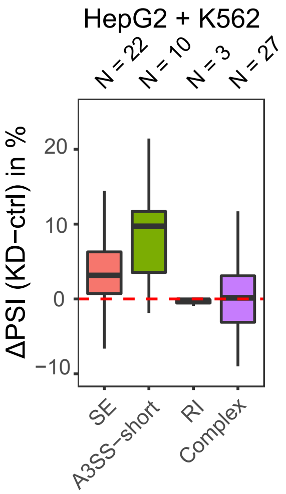

Supplemental Fig S11. A-to-I editing sites in splice site (ss) regions and associated splicing changes upon ADAR1 knockdown (KD) in HepG2 and K562 cells. RNA-Seq data were obtained from the ENCODE consortium. (A) Similar as Fig. 3A, difference in maxEnt scores of splicing sites harboring G or A at the indicated positions. (B) Similar as Fig. 3B, PSI change comparing ADAR1 KD vs. control cells for exons with editing sites in the 3'ss (changing AG to GG, i.e., 3'ss.2i). Only exons with adequate coverage (see Methods) in the ADAR1 KD and control RNA-Seq data for PSI calculation were included. Since the number of exons in each cell line was small, we combined the data from the two cell lines.
