# Supplementary material for: RNA editing in nascent RNA affects pre-mRNA splicing
Source: Genome Res. 2018 Jun;28(6):812–23. doi: 10.1101/gr.231209.117 (PMC5991522; doi:10.1101/gr.231209.117)
Supplement: Supplemental Material [file supp_gr.231209.117_Supplemental_Fig_S12.pdf]

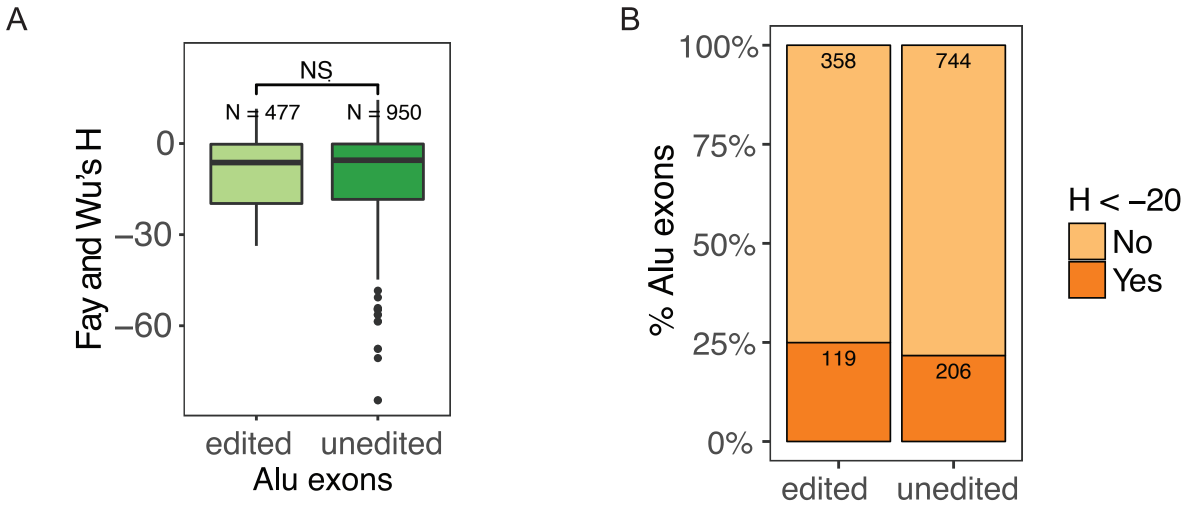

Supplemental Fig S12. (A) Distribution of H values in edited and unedited Alu exons. The editing sites can be anywhere in the Alu exons. N: number of exons. NS: P > 0.05, Wilcoxon rank sum test. (B) Proportions of Alu exons with H < -20, which previous literature used to define positively selected regions (Pandey et al. 2016), vs. otherwise. There was no significant difference in the number of exons with H < -20 (Fisher's exact test, P = 0.18). The number in the bars represent the number of exons.
