# Supplementary material for: RNA editing in nascent RNA affects pre-mRNA splicing
Source: Genome Res. 2018 Jun;28(6):812–23. doi: 10.1101/gr.231209.117 (PMC5991522; doi:10.1101/gr.231209.117)
Supplement: Supplemental Material [file supp_gr.231209.117_Supplemental_Fig_S13.pdf]

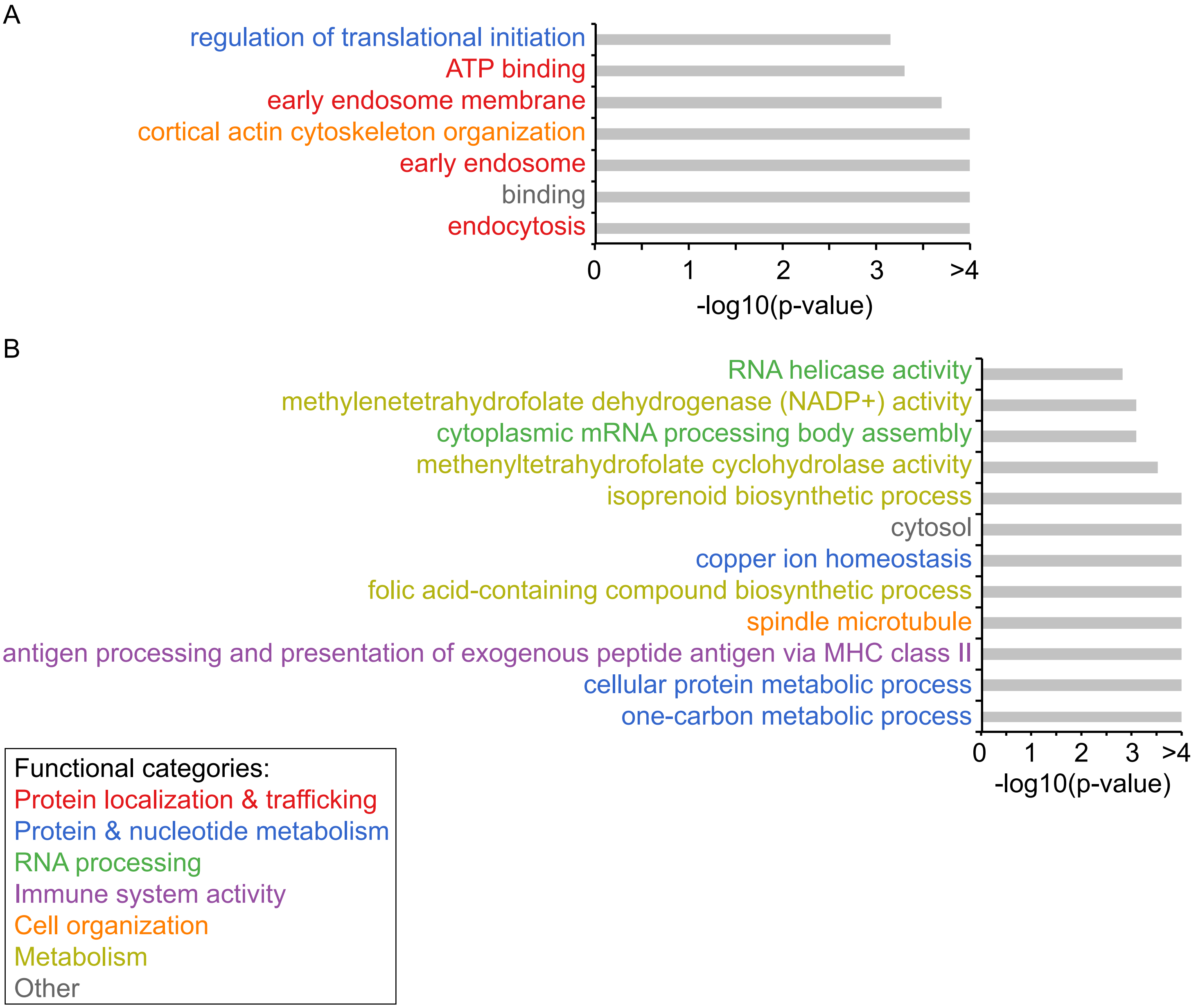

Supplemental Fig S13. GO analysis of genes that harbor editing sites in the 3’ss (AG to GG). GO categories corresponding to different types of biological functions are color-coded (see Box). (A) Enriched GO terms among genes that contain Alu-overlapping exons with 3’ss editing. For this analysis, the background control genes consisted of those that have Alu-overlapping exons but without editing sites in their 3’ss. (B) Enriched GO terms among genes that contain non-Alu-overlapping exons with 3’ss editing. The background control genes were those that have non-Alu-overlapping exons but without editing sites in the 3’ss.
