# Supplementary material for: RNA editing in nascent RNA affects pre-mRNA splicing
Source: Genome Res. 2018 Jun;28(6):812–23. doi: 10.1101/gr.231209.117 (PMC5991522; doi:10.1101/gr.231209.117)
Supplement: Supplemental Material [file supp_gr.231209.117_Supplemental_Fig_S14.pdf]

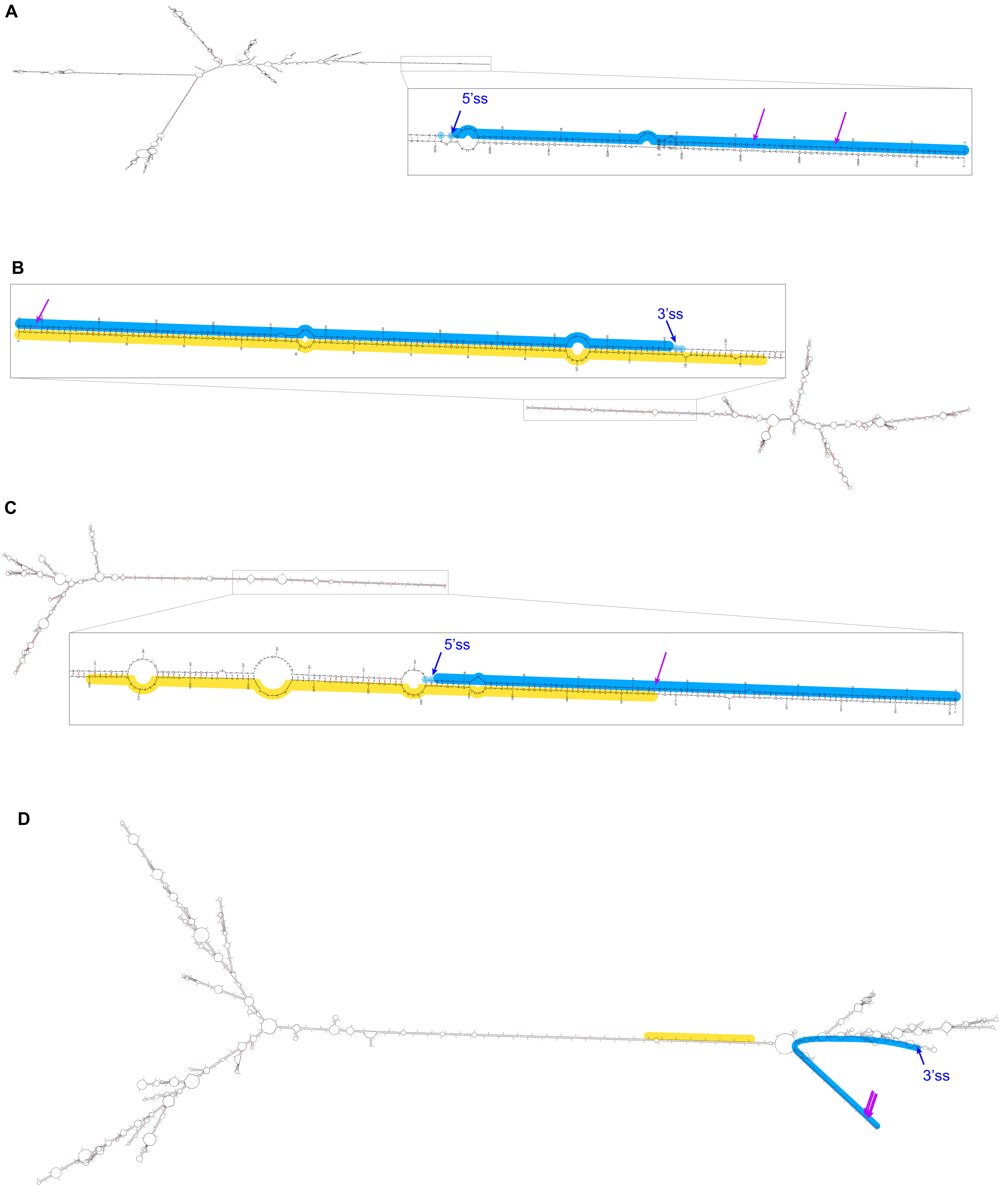

Supplemental Fig S14. RNA secondary structures predicted by mfold for each experimental candidate. (A) *LIPT1*. (B) *RFWD3*. (C) *EP400NL*. (D) *C17orf67*. Regulated exons are labeled in blue. CLIP peaks are labeled in yellow. Blue and purple arrows mark the splice site of regulated exons and editing sites, respectively.
