# Supplementary material for: RNA editing in nascent RNA affects pre-mRNA splicing
Source: Genome Res. 2018 Jun;28(6):812–23. doi: 10.1101/gr.231209.117 (PMC5991522; doi:10.1101/gr.231209.117)
Supplement: Supplemental Material [file supp_gr.231209.117_Supplemental_Fig_S15.pdf]

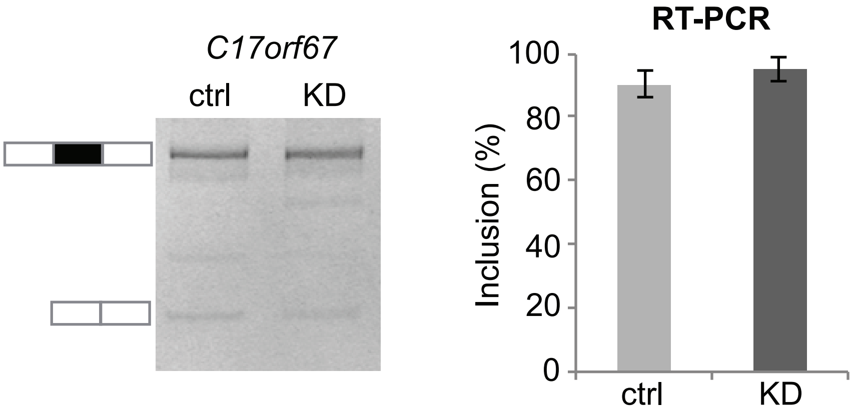

Supplemental Fig S15. Experimental test of the splicing pattern of a 3’ss-edited exon in the gene *C17orf67*. Exon skipping of the black middle exon was observed in the gel (left image), but with additional bands that indicate possible non-specific primers or complex splicing patterns. For the two splicing isoforms indicated next to the gel image, insignificant difference in exon inclusion levels was detected between ADAR1 KD and control U87MG cells (right panel).
