# Supplementary material for: RNA editing in nascent RNA affects pre-mRNA splicing
Source: Genome Res. 2018 Jun;28(6):812–23. doi: 10.1101/gr.231209.117 (PMC5991522; doi:10.1101/gr.231209.117)
Supplement: Supplemental Material [file supp_gr.231209.117_Supplemental_Methods.pdf]

Supplemental Material

A-to-I RNA editing in nascent RNA affects pre-mRNA splicing

By Hsiao et al.

Supplemental Methods
Supplemental References
Supplemental Figures 1-15
Supplemental Tables 1-6 (in Excel file)
Supplemental_Code.zip

Supplemental Methods

Percent sequence identity analysis

As a measure of sequence conservation, we calculated the percent sequence identity of genomic sequences ±100bp around the editing sites of interest. Percent sequence identity was defined as the number of aligned and identical nucleotides normalized by the total number of nucleotides. Since most editing sites were located in Alu sequences, we restricted this analysis to 11 primate genomes (Human, Chimp, Gorilla, Orangutan, Rhesus, Baboon, Marmoset, Tarsier, Mouse lemur, Bushbaby, and Tree shrew). The genomic alignments of these primates were extracted from the 46-way multiz alignments downloaded from the UCSC Genome Browser (Kent et al. 2002).

PhastCons analysis of non-Alu exons

To evaluate the conservation level of editing-dependent splicing events in non-Alu regions, we focused on the exon skipping events identified from U87MG, HepG2, or K562 ADAR1 KD data. As controls, we used the annotated human skipped exons curated by MISO (Katz et al. 2010). For both editing-dependent splicing events and controls, we calculated the PhastCons scores of 46 vertebrate genomes downloaded from the UCSC Genome Browser (Siepel et al. 2005; Kent et al. 2002).

SNP density in Alu-overlapping exons

For Alu-overlapping exons with editing sites in the 3’ss (AG to GG), we calculated the SNP density within the exons to evaluate their sequence preservation in the human population. Common SNPs from dbSNP version 150 were downloaded from UCSC Genome Browser (Kent et al. 2002). SNP density was calculated as the number of SNPs in the exon normalized by the length of the exon.

Gene ontology (GO) analysis

We adapted our previous approach (Lee et al. 2011) to identify enriched GO terms in genes harboring editing-dependent splicing events. For each target gene in our list, we chose a random control gene with exons in the same type of repetitive region (Alu or non-Alu) that do not have editing sites in the 3'ss. The gene length and GC content of control genes were required to differ by less than 5% relative to those of the corresponding target genes. We then calculated the frequency of occurrence of each GO term in the control set (denoted as X). This process was repeated 10,000 times to generate an empirical frequency distribution for each term, and an empirical P-value was calculated as P = Pr ($X \geq x$), where x denoted the number of occurrence of a term in the target gene list. The significance cutoff for choosing enriched terms was 1/total terms considered.

Production of lentivirus and cell transduction

pLKO1 non-target control-shRNA (SHC016) and ADAR1-targeting shRNA (TRCN0000050789) constructs were generous gifts from Dr. Gene Yeo (University of California, San Diego). We produced lentiviruses via co-transfection of pCMV-d8.91, pVSV-G and pLKO1 into HEK293T cells using Lipofectamine 3000 (Thermo Fisher Scientific, Cat# L3000-008). Transduction was carried out according to the standard protocol of the ENCODE consortium (Sundararaman et al. 2016). Briefly, viruses were collected from conditioned media after 48hr co-transfection. Lentivirus-containing media was mixed with the same volume of DMEM media that contain polybrene (8 μg/ml), which was used to infect U87MG cells. After 24hrs, cells were incubated with puromycin (2 μg/ml) for 3-7days. Knockdown efficiency was evaluated by Western blot using ADAR1 antibody (Santa Cruz Biotech, Cat# sc-73408) and beta-actin antibody (Santa Cruz Biotech, Cat# sc-47778 HRP).

Transfection, RNA extraction, reverse transcription, and PCR

Twelve-well HEK293 cells (80% confluence) were transfected with 1 μg minigene construct using Lipofectamine 2000 (Thermo Fisher Scientific, Cat# 11668019). HEK293 cells were

harvested 24 h post-transfection and total RNA was subsequently extracted using the TRIzol method (Thermo Fisher Scientific, Cat# 10296028). Two microgram total RNA was reverse transcribed into cDNA by ProtoScript® II Reverse Transcriptase (NEB, Cat#M0368L). One twentieth of cDNA was used as template for PCR (25 cycles). The reactions, in a total volume of 20 µL, consisted of 10 µL of One*Taq* 2X Master Mix with Standard Buffer (NEB, Cat#0482L), 1 µM of each minigene RT-PCR primer, and 1 µL of cDNA product. The cDNA was first denatured by heating at 95°C for 30 s, followed by 25 cycles at 94°C for 18 s, 60 °C for 20 s, and 68°C for 25 s. A final extension was done at 68°C for 5 min.

Gel electrophoresis and quantification

Five microliter of PCR product was loaded onto 5% or 10% polyacrylamide gel and electrophoresis at 70 volt for one and a half hours. The gel was then stained with SYBR® Safe DNA Gel Stain (Thermo Fisher Scientific, Cat# S33102) for half an hour before imaging via Syngene SYBRsafe program (Syngene). Expression levels of spliced isoforms were estimated using the ImageJ software (http://imagej.nih.gov/ij/). Inclusion rate (% inclusion) of the target exon was calculated as the intensity ratio of upper/(upper+lower) bands.

Supplemental References

Katz Y, Wang ET, Airoldi EM, Burge CB. 2010. Analysis and design of RNA sequencing experiments for identifying isoform regulation. *Nat Methods* **7**: 1009–15.

Kent WJ, Sugnet CW, Furey TS, Roskin KM, Pringle TH, Zahler AM, Haussler a. D. 2002. The Human Genome Browser at UCSC. *Genome Res* **12**: 996–1006.

Lee J-H, Gao C, Peng G, Greer C, Ren S, Wang Y, Xiao X. 2011. Analysis of transcriptome complexity through RNA sequencing in normal and failing murine hearts. *Circ Res* **109**: 1332–41.

Siepel A, Bejerano G, Pedersen JS, Hinrichs AS, Hou M, Rosenbloom K, Clawson H, Spieth J, Hillier LW, Richards S, et al. 2005. Evolutionarily conserved elements in vertebrate, insect, worm, and yeast genomes. *Genome Res* **15**: 1034–50.

Sundararaman B, Zhan L, Blue SM, Stanton R, Elkins K, Olson S, Wei X, Van Nostrand EL, Pratt GA, Huelga SC, et al. 2016. Resources for the Comprehensive Discovery of Functional RNA Elements. *Mol Cell* **61**: 903–913.
